# Genome‐wide association studies reveal genetic control of nutritional quality, milling traits, and agronomic characteristics in oat (*Avena sativa* L.)

**DOI:** 10.1002/tpg2.70060

**Published:** 2025-07-07

**Authors:** Naa Korkoi Ardayfio, Christy Kinney, Dandan Li, Sepehr Mohajeri Naraghi, Michael S. McMullen, Jason D. Fiedler

**Affiliations:** ^1^ Department of Plant Sciences North Dakota State University Fargo North Dakota USA; ^2^ USDA‐ARS Cereals Crops Improvement Research Unit Edward T. Schafer Agricultural Research Center Fargo North Dakota USA

## Abstract

Marker‐trait associations (MTAs) are invaluable to the understanding of biological processes and implementation of marker‐assisted selection (MAS) to increase genetic gain in modern breeding programs. In this study, a genome‐wide association study (GWAS) was performed to identify MTAs that influence oat nutritional quality, as well as agronomic and milling traits, in advanced breeding spring oat (*Avena sativa* L.) germplasm from North Dakota State University. High‐density sequence‐based molecular markers (15,037) were identified in a population of 1,092 unique lines evaluated in 11 field conditions over three years. Linkage disequilibrium analysis confirmed that decay metrics of 5, 11.5, and 2.2 Mb for the A, C, and D sub‐genomes, respectively, matched the 1.25 Mb/single nucleotide polymorphism marker density used in this study. We identified 160 MTAs and 44 durable quantitative trait loci (QTL) for nine traits that individually explained 3.7%–43.9% of the variation in the data. Haplotypes assembled from the three most predictive QTL for each trait increased the variation explained to 16%–46%. The haplotype effects are substantial, with their presence resulting in predicted mean percentage increases—greater than 20% for many of the investigated traits. These results confirm that significant MTAs for oat can be identified directly within breeding germplasm using GWAS. These markers are excellent candidates for the implementation of MAS to increase the selection efficiency and genetic gain.

## Core Ideas

- A genome‐wide association study was conducted on advanced germplasm of an oat breeding program.
- Several marker‐trait associations (MTAs) were found for breeding‐related traits.
- Haplotype analysis of the most important MTAs highlights their potential for early screening.

**Abbreviations**
AYT: advanced yield trial
BLUE: best linear unbiased estimator
GWAS: genome‐wide association study
LD: linkage disequilibrium
MTA: marker‐trait association
NDSU: North Dakota State University
PYT: preliminary yield trial
QTL: quantitative trait locus
SNP: single nucleotide polymorphism

## 1 INTRODUCTION

Oat (*Avena sativa* L.) is an important cereal crop worldwide. In the United States, oat, which requires minimal inputs, is commonly produced as a grain or forage in an annual rotation. In 2022, over 1 million ha of planted oat yielded 57 million bushels of oat grain (groat) and generated over $294 million in revenue (USDA‐NASS, 2020). Additionally, oat cultivation has been shown to improve soil health and can increase the yields of rotation crops grown afterward (Bowles et al., 2020).

Oat possesses a unique nutritional profile of essential amino acids, fatty acids, dietary fibers, and phenolic compounds (Butt et al., 2008; Strychar, 2011; Peterson et al., 1992), making it an exceptionally healthy grain for human consumption. Among these nutritional qualities, macronutrients β‐glucan, oil, and protein have been prominent foci for breeding programs. Cereal β‐d‐(1→3), (1→4)‐glucan, most abundant in oat and barley, is a hemicellulose present in the cell walls of the endosperm (Fincher, 2009). Consumption of this dietary fiber reduces blood pressure, lowers serum cholesterol levels, and improves immune response—which in turn decreases the risk of cardiovascular disease, diabetes, and cancer (Braaten et al., 1994; Estrada et al., 1997; Grundy et al., 2018; Jenkins et al., 2002; Keenan et al., 2002; Meydani, 2009). Notably, in scientific summary [21 Code of Federal Regulations (CFR) 101.9(c)(6)(i) and 21 CFR 101.81], the US Food and Drug Administration has confirmed that consumption of oat β‐glucan can reduce coronary heart disease risk and has approved health benefit labeling on oat products containing a minimum of 4.5% β‐glucan content (FDA, 2018).

Oat oil content provides significant health benefits for consumers. Healthy unsaturated oleic and linoleic fatty acids are highly concentrated in oat oil, which can comprise up to 18% of the edible grain (Brown & Craddock, 1972; Brown et al., 1966; Halima et al., 2015). Unsaturated fatty acids have been shown to reduce low‐density lipoprotein cholesterol in the blood, consequently reducing coronary heart disease risk (Mensink & Katan, 1992). Oil content is a highly heritable trait influenced by multiple genes with additive effects (Thro & Frey, 1985). Breeding goals for oat oil content are bifurcated, with low‐oil varieties considered desirable for milled products and high‐oil varieties showing promise as ingredients in novel food ingredients and cosmetics (Banaś & Harasym, 2021).

The protein composition and abundance in oat has revealed its potential as a significant plant‐based dietary protein source. Protein levels can exceed 20% by weight (Sunilkumar et al., 2017) and are composed of mostly non‐immunogenic globulin and avenin classes, accounting for 50%–80% and 10%–20% of the total grain protein, respectively (Frey, 1951; Peterson & Smith, 1976). Thus, oats can be consumed without the allergy and tolerance concerns posed by soy and wheat protein sources.

Given its numerous health benefits and food product potential, oat is well‐positioned to meet the nutritional needs of the ever‐growing world population. However, in the face of emerging diseases and variable growing conditions, new oat varieties must continually be released to reliably provide high amounts of quality grain. The development of new oat cultivars has traditionally been conducted through direct phenotypic selection. With this method, breeders aim to simultaneously improve many agronomic, disease resistance, quality, and yield component characteristics to make the crop appealing for producers, processors, and consumers (Murphy & Hoffman, 1992). Due to the inverse correlations of many traits, both parent and cultivar selections can be challenging when employing a single breeding selection strategy (Lewis, 2006; W. Yan & Wallace, 1995). Additionally, it takes several site‐years of evaluations after progeny have been sufficiently inbred to determine if a particular line is promising enough for parent selection or promotion to variety candidate trials. For disease and abiotic stress resistance, introgression from wild progenitors has been employed to transfer these qualitative traits to adapted germplasm (Huang & Han, 2014). However, this process is laborious due to the low efficiency of interspecific crosses in the *Avena* genus and the significant resources required to break linkage drag.

Molecular breeding tools use predictive genomic loci to improve the efficiency of trait selection and introgression, enabling more rapid development of new cultivars compared to phenotypic selection (Bevan & Uauy, 2013). Marker‐assisted selection (MAS) uses single nucleotide polymorphisms (SNPs) or small insertion/deletion (InDel) markers that are genetically linked to quantitative trait loci (QTL) to enable the prediction of traits without direct observation. With high‐throughput screening of these marker‐trait associations (MTAs) in early‐generation germplasm, desirable traits can be enriched in lines that are evaluated directly for selection (Tanksley et al., 1989; Young, 1999).

Genome‐wide association studies (GWASs) are a set of widely used procedures that connect phenotypic data and high‐density genotypic data to identify QTL and linked MTAs across the genome. This approach utilizes historical recombination in nonstructured populations and can capture large amounts of variation within crops (Hamblin et al., 2011). When genomes are saturated with molecular markers, gene‐level resolution mapping can be achieved (Brachi et al., 2011). Through GWAS, MTAs have been identified for various important traits in many crops, including oat. One such study, using 446 North American oat breeding lines genotyped with 1,005 Diversity Array Technology markers and two years of phenotypic data, identified 21 molecular markers that influenced β‐glucan concentration with a single marker test. Application of the mixed‐model least absolute shrinkage and selection operator with the same data identified 51 significant markers, 15 of which had been previously reported (Asoro et al., 2013). Newell and colleagues also conducted a GWAS with 431 oat accessions from the National Small Grains Collection. Three independent markers were identified as significantly associated with groat β‐glucan content. One of these significant markers showed homology to a genetic sequence on rice Chr7 adjacent to the CslF gene family that has been implicated with β‐glucan synthase function (Newell et al., 2012). In another GWAS, field experiments were conducted with 174 varied oat accessions to evaluate groat protein content (GPC) in three locations. Of the 38,313 genotyping‐by‐sequencing markers identified in the study, 41 markers tagged 27 unique QTL that influence GPC. Two QTL on chromosomes 6C and 4D, were highly predictive and detected in multiple environments (Yan et al., 2023). A GWAS study was conducted on a total of 2,993 lines from a nested association mapping population of 10 diverse spring oat lines where 60 unique variables for panicles, rachis, and spikelets were investigated. The authors discovered many QTL and concluded that taller, stiff stalked and open panicle structured oat plants were important for adult plant resistance for crown rust (Carlson et al., 2023) As a final example, three different GWAS approaches (multivariate mixed model approach, principal component, and univariate analyses) were used to evaluate the composition of ten fatty acids in 500 oat lines grown in two field environments. These methods identified 148, 129, and 73 significant MTAs (Carlson et al., 2023).

**Core Ideas**

- A genome‐wide association study was conducted on advanced germplasm of an oat breeding program.
- Several marker‐trait associations (MTAs) were found for breeding‐related traits.
- Haplotype analysis of the most important MTAs highlights their potential for early screening.

The use of unbalanced data in GWAS to identify QTL has become increasingly popular due to the availability of many years of experimental trials from breeding programs that often use this field design to increase efficiency. The predominant drawbacks of an unbalanced data set for genetic studies are limited population sizes and the increased risk of false positives. A study was conducted on barley breeding germplasm by Wang et al. (2012) to evaluate differences between a balanced and unbalanced population in detecting QTL. Two panels were created to evaluate heading date, which comprised of a balanced trial of 766 spring barley breeding genotypes and a second panel of a balanced and unbalanced trial subset of 384 spring barley breeding genotypes. Three QTL that had been previously detected in a biparental mapping studies were identified in both panels. There was, however, an increase in false‐positive rate in the unbalanced dataset, which was reduced in a one‐step analysis. The researchers concluded that data from breeding programs are important to detect QTL, but experimental design and population size must be carefully considered (Wang et al., 2012).

In this study, we conducted GWAS with late‐stage breeding germplasm from the North Dakota State University (NDSU) breeding program that was evaluated over three years in multiple field sites. The overall objectives of the study were to (i) identify predictive QTL for agronomic, milling, and nutritional quality characteristics in hexaploid oats, (ii) select the most informative markers for each QTL, and (iii) predict trait outcomes with haplotype analysis of unlinked QTL. With this knowledge in hand, we aim to accelerate breeding efforts with the conversion of QTL into high‐throughput informative markers and screening of early‐generation germplasm‐.

Molecular breeding methods such as MAS and genomic selection (GS) are both powerful tools to increase genetic gain in breeding programs (Goddard & Hayes, 2007; Jannink et al., 2010; Kou & Wang, 2010; Weckwerth, 2011). They both use molecular markers to predict traits, but differ in the scope of the genotyping and processing of the decision support model for the breeder. Single or multiple large‐effect QTL that reliably predict traits can be quickly ascertained with low‐cost polymerase chain reaction‐based markers and directly converted to a trait score in an MAS scheme. On the other hand, GS can better predict highly quantitative traits that result from many low‐effect QTL but is more expensive to implement as it requires a more costly durable genome‐wide marker platform and a relatively complex statistical model. Here we focus on supporting MAS through the identification of a small set of high‐effect QTL because of its cost and ease of use in many classical breeding programs that have not utilized molecular breeding tools in the past.

## 2 MATERIALS AND METHODS

### 2.1 Plant materials

The 1092 unique NDSU breeding lines used in this study were evaluated in North Dakota in 2016, 2018, and 2019. There were several related parents within the breeding lines, as shown by pedigree in Tables S1 and S2. Certain check genotypes were repeated across years and experimental trials (Table S3). Breeding lines in the seventh generation of self‐pollination were evaluated in preliminary yield trials (PYTs) in 2016. Evaluation in 2018 and 2019 consisted of PYT lines in the seventh‐generation and eighth‐generation lines from advanced yield trials (AYT). For each trial, lines were evaluated in 4 × 8 feet plots in a randomized complete block field design with each replicate representing a block. The middle two rows of each block were harvested to ensure genotype purity during pollination. The 2016 trials were conducted in two locations with two replications per location (Table 1). Traits measured in 2016 included heading date (days to head), height (cm), thins (%), yield (kg/ha), and test weight (kg/hL). The 2018 trials consisted of five experiments: two AYTs with four locations and three PYTs with two locations. Experimental trials PYTs and AYTs had different experiments that focused on different breeding objectives. For instance, there were early, mid, and late maturing experiments. The number of experiments was consistent between locations of the same year. Entries within experiments were replicated three times in AYTs and twice in PYTs. Entries denotes numbers assigned to individual genotypes in a replication. A second replication of the same location will therefore consist of a randomization of the individual genotypes (entries) of the first replication in the same location. Traits measured in 2018 included β‐glucan (%), protein (%), oil (%), heading date, height, test weight, thins, yield, and groat (%). In 2019, there were four PYT experiments grown in two locations with two replicates each, as well as two AYT experiments grown in five locations with three replicates each. Traits measured in 2019 included β‐glucan, protein, oil, heading date, height, test weight, thins, yield and groat. The number of locations, traits measured, and replications in PYTs differed from that of AYTs. PYTs across the three years consisted of the Casselton and Fargo locations, each with two replications. The AYTs of 2018 and 2019 consisted of four and five locations respectively, with three replications each. The specific experimental trials across the three years and their unique genotype number, together with locations, traits measured within location and between years as well as specific replications within location and years is tabulated in Table S1. Field evaluation locations were near the North Dakota cities of Fargo (DMS Latitude: 46° 52′ 37.0596″ N, DMS Longitude: 96° 47′ 4.6932″ W), Casselton (DMS Latitude: 46° 54′ 1.8036″ N, DMS Longitude: 97° 12′ 40.8852″ W), Carrington (DMS Latitude: 47° 26′ 58.99″ N, DMS Longitude: 99° 07′ 34.41″ W), Edgeley (DMS Latitude: 46° 21′ 37.29″ N, DMS Longitude: 98° 42′ 43.38″ W), Verona (DMS Latitude: 46° 21′ 49.89″ N, DMS Longitude: 98° 04′ 20.36″ W), and Minot (DMS Latitude: 48° 13′ 48.5904″ N, DMS Longitude: 101° 17′ 28.3164″ W).

**TABLE 1:** Summary of spatially adjusted trait measurements for each location per year and averaged trait best linear unbiased estimator (BLUE) values.

Year/location	β ‐Glucan (%)	Protein (%)	Oil (%)	Test weight (kg/hL)	Groat (%)	Yield (kg/ha)	Thins (%)	Heading date (days)	Height (cm)
**2016**	–	–	–	**50 ± 2.6**	–	**4878 ± 738.9**	**9 ± 4.2**	**23 ± 3.3**	**99 ± 12.1**
Casselton	–	–	–	50 ± 2.5	–	5309 ± 677.9	9 ± 4.2	25 ± 2.2	109 ± 7.4
Fargo	–	–	–	50 ± 2.6	–	4448 ± 520.1	9 ± 4.3	20 ± 2.0	89 ± 6.1
**2018**	**5 ±** **0.5**	**17 ± 1.2**	**6 ± 0.7**	**51 ± 2.2**	**74 ± 4.4**	**4878 ± 764.0**	**8 ± 3.6**	**23 ± 1.8**	**94 ± 7.7**
Carrington	–	–	–	55 ± 2.1	–	4663 ± 573.9	9 ± 4.2	–	–
Casselton	–	–	–	51 ± 1.7	–	5309 ± 835.8	7 ± 3.5	–	100 ± 5.0
Edgeley	–	–	–	54 ± 1.6	–	4197 ± 491.4	8 ± 3.6	–	–
Fargo	5 ± 0.5	17 ± 1.2	6 ± 0.7	50 ± 2.0	74 ± 4.4	4555 ± 444.8	7 ± 3.5	23 ± 1.8	89 ± 5.2
**2019**	**4 ± 0.5**	**14 ± 1.3**	**6 ± 0.8**	**50 ± 2.4**	**74 ± 4.8**	–	**7 ± 3.7**	**38 ± 2.7**	**111 ± 9.0**
Carrington	4 ± 0.5	14 ± 1.2	6 ± 0.7	51 ± 1.4	76 ± 2.0	–	6 ± 2.2	–	–
Casselton	4 ± 0.5	14 ± 1.3	6 ± 0.8	‐	74 ± 5.3	–	6 ± 3.6	–	115 ± 8.5
Fargo	–	–	–	‐	–	–	7 ± 3.9	38 ± 2.7	106 ± 7.1
Minot	–	–	–	51 ± 1.7	–	–	10 ± 3.8	–	–
Verona	–	–	–	47 ± 1.7	–	–	7 ± 3.5	–	–

*Note*: Values are averages ± standard deviation, Bold values denote year BLUEs.

### 2.2 Phenotyping

Experiments were conducted under field conditions at the various experiment stations. Soil amendments were added when necessary to maintain optimum plant growth according to standard local management practices. Agronomic and milling traits measured include test weight, groat percentage, proportions of both plump and thin grains per sample, heading date, and height. Individual traits were measured in various years and locations as specified in Table 1. Heading date was evaluated when 50% or more panicle nodes had just emerged from the flag leaf sheath and recorded as the number of days after May 31. Height measurements were collected when the oat plants began senescence and were measured in centimeters from the ground level to the tip of the longest panicle, guided by the hand along a measuring pole. Quality and yield‐related traits were measured after harvest. Oat panicles were threshed in the field, and grains were weighed for yield estimate after 14% moisture was obtained. Test weight, which represents grain density, was measured by weighing grain contained in a 500‐mL chondrometer and reported in kilograms per hectoliter, with higher test weight indicating higher quality grain. Plumps and thins are subsets of yield in which thins represent the smallest seed sizes, typically from the tertiary florets, while plumps are the large seeds, typically from the primary and secondary florets, that meet a desirable milling threshold value. These subsets were determined after threshing by sifting grains with a 5/64 inch (12.7 cm) perforated screen. Grains ≤5/64 inch (12.7 cm) were considered thins, and presented as a percentage of the total grain sample.

Oat groats are the portion of grains (kernels) left after removal of the outer hull. Samples were de‐hulled in a Codema dehuller for 1 min 30 s duration, and groat percentage was calculated from groat weight as a fraction of the entire weight of the whole grain. β‐glucan, protein, and oil content were measured using near infrared (NIR) spectroscopy on 50 g of ground or whole de‐hulled seed in an NIR grain analyzer and reported as a percentage of total mass. These phenotypic data used in this study are available in Table S2.

### 2.3 Genotyping‐by‐sequencing

Individuals from F_7_ generation of the NDSU breeding germplasm (*n* = 1,124) were genotyped with a previously described dual‐enzyme genotyping‐by‐sequencing (GBS) method (Carlson et al., 2023) with some minor modifications in the bioinformatic processing. Approximately 2.5 million paired end 2 × 150 bp reads per line were acquired for this population and after quality filtering and trimming with bbduk (BBTools, https://jgi.doe.gov/data‐and‐tools/bbtools), sequences were aligned to the *A. sativa* OT3098 reference genome assembly (v1; PepsiCo, 2021; https://wheat.pw.usda.gov/GG3/graingenes_downloads/oat‐ot3098‐pepsico) with Bowtie 2 (Langmead & Salzberg, 2012). Duplicate alignments were marked with *samblaster* (Faust & Hall, 2014) and reads with a mapQ score of at least 30 were processed with *samtools/bcftools* (Li, 2011) to identify population‐level SNPs and small insertions/deletions. Genotype calls were filtered with VCFtools (minDP = 2, max‐missing = 0.5, maf = 0.01) and TASSEL v 5.2.50 (Bradbury et al., 2007) to remove sites with greater than 10% heterozygous calls. Missing data were imputed with the LD‐KNNi method (Money et al., 2015), and an additional filtering step removed taxa with greater than 75% missing data or greater than 20% heterozygous calls. Positions in this file were then lifted over to the v2 release of the OT3098 genome (PepsiCo, 2021) using unambiguous BLAST alignments of the surrounding sequence. This final genotyping data contained 15,037 sites (13,348 SNPs; 1,689 InDels), 1,092 taxa, and contained an overall missingness rate of 2% and heterozygous call rate of 4.6%.

### 2.4 Statistical analysis

Linkage disequilibrium (LD) was calculated on a 50‐site sliding window with TASSEL5 (v5.2.50). The LD decay half‐life metrics were estimated for each chromosome, sub‐genome, and the entire genome using nonlinear regression of the *r* ^2^ value versus distance between sites (Abecasis et al., 2001; Hill & Weir, 1988) (Table 2) with a custom script in R (v4.2.1). Visualization of LD data was conducted with the TASSEL5 heatmap chart tool. Visualization of the trait data was conducted with ggpairs function in the GGally R package (Schloerke et al., 2022) and Adobe Illustrator. Multi‐environment trial data were analyzed using a two‐stage approach. In Stage 1, each trial was analyzed using a spatial model implemented in the SpATS R package (Rodríguez‐Álvarez et al., 2018). The spatial model was as follows:

$$Yijk=Gi+f\left(rk,cl\right)+Bj+Rk+Cl+\varepsilon ijk,$$

where *Yijk* is the observed phenotype, *Gi* is the effect of genotype *i*, *f*(*rk*, *cl*) represents the smooth spatial surface modeled using P‐splines, *Bj* is the fixed effect of block *j*, *Rk* and *Cl* are the random effects of row *k* and column *l*, respectively, and *εijk* is the residual error. The spatial surface was modeled using a penalized spline analysis of variance approach with tensor product P‐splines. Means were summarized in Table 1, listed in Table S4, and visualized in Figure S1.

**TABLE 2:** Linkage disequilibrium (LD) decay half‐life for individual chromosomes, sub‐genomes, and the entire genome.

LD decay (Mb)	Sub‐genome		
Chromosome	A	C	D
1	4.89	358.10	2.08
2	4.28	254.51	2.40
3	3.45	20.13	0.76
4	5.32	31.98	2.28
5	8.84	3.07	1.88
6	4.11	4.71	3.79
7	4.08	2.91	6.16
Total	4.99	11.47	2.21
Genome	6.11		

In Stage 2, spatially adjusted means were combined across environments using a weighted mixed model approach. First, within each environment, adjusted means were normalized using check varieties:

$$\mathrm{Normalized}\_ij=\hat{Y}ij/\mu c\_j,$$

where *Ŷij* is the adjusted mean of genotype *i* in environment *j*, and *μc*_*j* is the mean of check varieties in environment *j*. Standard errors were propagated through normalization as SE_norm = SE/*μc*_*j*. The across‐environment model was as follows:

Normalized_ij=μ+Gi+Ej+GEij+εij,

where *Gi* is the fixed effect of genotype *i*, *Ej* is the random effect of environment *j*, *GEij* is the random genotype‐by‐environment interaction, and *εij* is the residual error. Observations were weighted using *w* = 1/SE_norm^2^. When the mixed model indicated convergence issues, a reduced fixed‐effects model was fitted as follows:

Normalized_ij=μ+Gi+εij.

Environments with fewer than three observations or zero variance were excluded from analysis. Genotype reliability was calculated as 1 − [SE^2^/var(BLUE)]., where SE is the standard error of the genotype estimate, var(BLUE) is the variance of all genotype estimates, and BLUE is the best linear unbiased estimator. Normalized BLUE values can be found in Table S5.

GWAS was performed with the Genome Association and Prediction Integrated Tool (Lipka et al., 2012; Zhang et al., 2010) within R using the Bayesian‐information and linkage‐disequilibrium iteratively nested keyway (BLINK) (Huang et al., 2019) model for the normalized BLUE values with the check entries removed (Table S5). The first three principal components were included as covariates to control for population substructure (explains 16.5% of variation; Figure S2). For all traits, the BLINK model performed well with *QQ* and Manhattan plots provided for each trait in Figure S3. For each trait of interest, markers with a percent variation explained (PVE) greater than 3% and a Benjami–Hochberg adjusted *p*‐value < 0.05 were retained for further analysis (Table S6).

To simplify visualization and description of the markers and QTL identified when using the normalized BLUE values, traits were grouped by allele, and genotype calls were converted to a presence (+) or absence (−) of the SNP/QTL to demark the greater or lesser numeric value, respectively (Table S6). Heterozygous genotype calls were present as a minor allele state for many of the markers selected above. A Wilcoxon signed rank‐test was used to determine if trait values of the heterozygous state were significantly different than one of the homozygous calls for the purposes of assigning a QTL designation. In most cases, heterozygous states could be unambiguously assigned (e.g., β‐glucan:S4C_27965926; Figure S4). In situations where it was ambiguous (e.g., β‐glucan:S6D_289646620 or oil:S4C_21549747; Figure S4), heterozygous calls were converted to missing. The PVE by each significant marker identified by GWAS was estimated by fitting a simple linear model (lm) in R relating the allele call to the trait scores (normalized BLUE values or individual environment means) and extracting the adjusted *R* ^2^ value. The effect of each QTL was estimated as the difference between the means of the trait variables grouped by the QTL presence or absence. This difference was multiplied by the per‐environment averages of the check lines to estimate a tangible trait value increase for discussion. Markers that displayed PVE > 2% in at least half of the environments tested (Table S7) were kept for functional assessment. The local LD environment of each marker was assessed in TASSEL to determine an approximate QTL linkage block size of the remaining durable markers, and stepwise regression was used to identify the three most important QTL for each trait with the R package olsrr v0.6.0.9 (ols_step_best_subset function). The selected QTL were named with the convention Q*trait*.fgl‐*Chr*#. Visualization of trait distributions by QTL and haplotype was generated with R package ggplot2 (v 3.5.1). Comparative analysis of the QTL identified in this study with previously published QTL was possible with alignment of markers underlying both on the OT3098 v2 reference genome and Sang reference genome (Kamal et al., 2022). Genes located within the estimated LD were evaluated for functionality. These data can be found in Table S8.

## 3 RESULTS AND DISCUSSION

### 3.1 Trait location relationships

**FIGURE 1:**
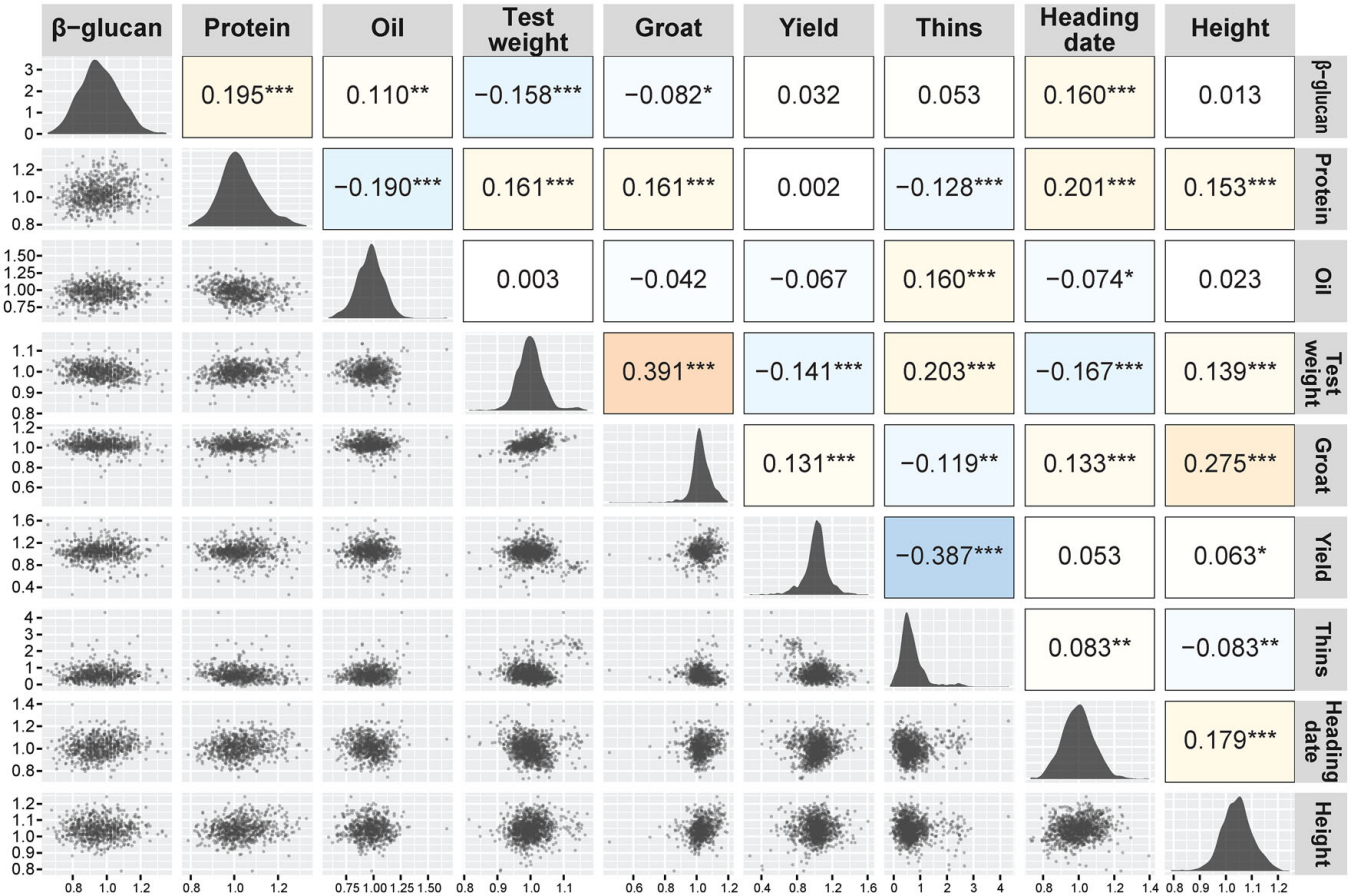
Pairwise correlation among trait best linear unbiased estimator (BLUE) values of β‐glucan, oil, protein, test weight, groat, yield, thins, heading date, and height. The diagonal displays the frequency distribution of the traits. The upper triangle shows the Pearson's correlation coefficient (*R*) between traits and the significance of correlation for the comparison (*p ≤ 0.05, **p ≤ 0.01, ***p ≤ 0.001). The lower triangle shows the scatterplots of the data.

In this study, trait data were collected over three years from up to six locations per year. Within the 11 year‐location combinations (environments) tested, there were differences among the trait distributions (Table 1; Figure S1), which can be attributed to the environment‐specific responses of the traits and the differences in germplasm grown in each environment. Due to the unbalanced nature of the data, spatially adjusted values within each experiment were normalized with up to 16 checks and combined into BLUEs (Tables S4 and S5). BLUEs were normally distributed for all traits except for thins, which were right‐skewed (Figure 1).

Pairwise comparisons among the trait BLUEs revealed relatively weak correlations, with the strongest inverse association found between thins and yield (*r* = −0.387) (Figure 1), which is expected as a lower thins value indicates a larger percentage of plump groat and greater mass per groat on average. Conversely, the strongest identified positive correlation was between test weight and groat (*r* = 0.391), which is indicative of a denser grain maintaining integrity during the milling process (Doehlert et al., 2006). The correlation between β‐glucan and protein BLUEs is consistent with the positive association of protein and β‐glucan reported in previous studies (Kibite & Edney, 1998; Peterson et al., 1995). Overall, the lack of strong correlations among these traits indicates a potential for the independent improvement of each trait through MAS.

### 3.2 Overall genetic analysis

The germplasm evaluated in this study originates from the NDSU oat breeding program. These lines were a combination of F_7_ and F_8_ lines derived from crosses made between 2007 and 2009. All the entries for the preliminary and advanced yield trials were genotyped with a sequence‐based protocol and after taxa‐based quality filtering for excessive data missingness and/or heterozygosity levels, 1,092 lines remained for association analysis.

After site‐based quality filtering, 13,349 SNPs and 1,689 InDel molecular markers were used for subsequent analysis. The average marker density of 1 per 1.25 Mb was less than the estimates of LD decay of 4.99, 11.47, and 2.21 Mb for the A, C, and D sub‐genomes, respectively (Table 2). The first three principal components cumulatively represented 16.5% of the genetic variation in the population, suggesting relatively low substructure, as well as suitability for GWAS (Figure S2).

### 3.3 Identified marker‐trait associations

Across the GWAS experiments conducted here, 158 MTAs were identified (Table S6) with 65 associations having an overall PVE greater than 3% and 44 of these displaying durable effects in multiple environments. Evaluation of the LD decay among 65 high‐effect markers enabled further binning into linkage blocks. The 44 durable markers evaluated here resided on 35 unique linkage blocks, with some blocks influencing multiple traits. This population displayed localized regions of high LD where large genomic blocks were inherited together with minimal recombination, especially near the centromeres. This was especially apparent for QProtein.fgl‐3A, which spanned ∼75% of the physical chromosome.

While 44 durable QTL were identified for the nine traits studied here, enrichment for all of them would be impractical in a breeding program. Therefore, stepwise regression was used to select the three most predictive QTL per trait (Table 3). These named QTL were evaluated alone and in concert as haplotypes to provide evidence for their combined usage to explain trait variation in this data set.

**TABLE 3:** Information about the three most predictive QTL and combined haplotype for traits.

QTL name	Chr	Position(s)	Size (Mb)	Linkage block (Mb)	TagSNP	TagSNP Chr (Sang)	TagSNP (Sang)	BinaryMin.Allele	BinaryMax.Allele	Min.Ave	Max.Ave	*R* ^2^ (%)	Haplotype *R* ^2^ (%)	Haplotype diff
Qβglucan.fgl‐6D	6D	289,646,620	12.6	279–291.6	S6D_289646620	chr6D	280274759	G	A	0.95	1.09	4.2	10.9	0.27
Qβglucan.fgl‐4C	4C	27,965,926	57.8	2–59.8	S4C_27965926	chr4C	36304705	A/R	G	0.94	0.98	3.9		
Qβglucan.fgl‐3A	3A	105,822,640	338.5	64.5–403	S3A_105822640	chr3A	99356317	G	T/K	0.95	1.01	3.7		

QProtein.fgl‐2A	2A	398,107,713	1.3	397.5–398.8	S2A_398107713	chr2A	391838360	G	A/R	1.01	1.06	6.1		
QProtein.fgl‐5A	5A	339,889,539	96	326–422	S5A_339889539	chr5A	341585243	G/R	A	0.98	1.04	5.8	13.9	0.16
QProtein.fgl‐3C	3C	580,559,480	516	90–606	S3C_580559480	chr3C	516556095	T	K	1.02	1.12	4.2		

QOil.fgl‐4C	4C	21,549,747	57.8	2–59.8	S4C_21549747	chr4C	29613037	G	A	0.91	1.01	16.9	28.3	0.24
QOil.fgl‐6A	6A	423,631,188	0.0003	–	S6A_423631188	chr6A	419213421	T/Y	C	0.86	0.98	11.0		
QOil.fgl‐5A	5A	469,061,327	–	–	S5A_469061327	chr5A	462151006	C	del/0	0.94	1.01	7.3		

QTestweight.fgl‐4D	4D	452,015,611	3.3	452–455.3	S4D_452015611	chr4D	415009851	C	T/Y	1.00	1.07	14.1	20.8	0.17
QTestweight.fgl‐6C	6C	312,911,424	242	246–488	S6C_312911424	chr6C	288842472	C/S	G	1.008	1.11	10.9		
QTestweight.fgl‐7A	7A	255,608,481	252.1	233.9–486	S7A_255608481	chr7A	253792761	T	C	0.95	1.00	4.0		

QYield.fgl‐4D.1	4D	455,312,110	3.3	452–455.3	S4D_455312110	chr4D	411462465	T	C/Y	0.78	1.04	16.7	20.9	0.34
QYield.fgl‐6C	6C	259,051,881	242	246–488	S6C_259051881	chr6C	243191511	T	C	0.68	1.03	7.0		
QYield.fgl‐4D.2	4D	450,577,160	0.6	450.6–451.2	S4D_450577160	chr4D	407603698	G	A	0.91	1.03	3.8		

QThins.fgl‐4D.1	4D	454,967,583	3.3	452–455.3	S4D_454967583	chr4D	411984450	G	A/R	0.6	2.02	43.9	45.9	1.99
QThins.fgl‐4D.2	4D	426,900,796	0.2	426.8–427	S4D_426900796	chr4D	384063720	A/R	G	0.61	2.1	34.2		
QThins.fgl‐6C	6C	24,305,773	64.5	22.6–87.1	S6C_24305773	chr6C	22479044	G	A	0.65	1.61	7.2		

QHeadingdate.fgl‐6A	6A	410,925,381	51.7	363–414.7	S6A_410925381	chr6A	407174339	C	T/Y	0.98	1.04	8.2	16.0	0.15
QHeadingdate.fgl‐6C	6C	103,114,557	13.1	102.9–116	S6C_103114557	chr6C	97233736	C	T	0.95	1.01	4.0		
QHeadingdate.fgl‐1D	1D	349,564,707	3.2	347.5–350.7	S1D_349564707	chr1D	339995569	C/Y	T	0.97	1.01	5.5		

QHeight.fgl‐6D	6D	279,296,541	13.5	278–291.5	S6D_279296541	chr6D	270768735	C	0/del	1.03	1.06	7	15.9	0.08
QHeight.fgl‐6A	6A	54,027,913	191.4	52.4–243.8	S6A_54027913	chr6A	54620180	A	G/R	1.03	1.06	6.0		
QHeight.fgl‐4D	4D	367,447,171	37	333–370	S4D_367447171	chr4D	328541356	C	0/del	1.03	1.06	5.5		

Abbreviations: Chr, chromosome; QTL, quantitative trait loci; SNP, single nucleotide polymorphism.

### 3.4 QTL for β‐glucan of hexaploid oat

**FIGURE 2:**
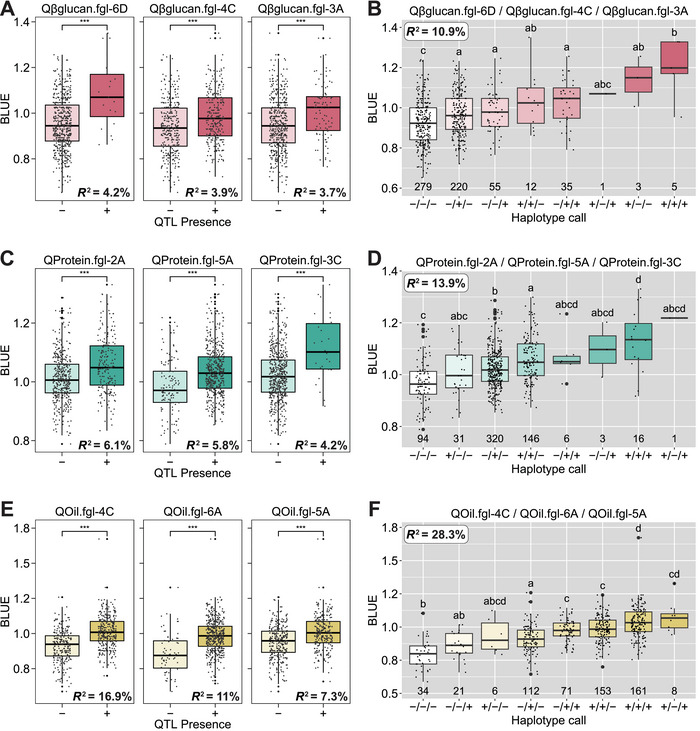
Distributions of three most useful quantitative trait loci (QTL) indicate significant differences using best linear unbiased estimator (BLUE) value with or without alleles of the specific markers of (A) β‐glucan content, (C) protein content, and (E) oil content. The significance of differences between the pairwise means was determined with the Wilcox test (***p ≤ 0.001). Haplotype calls of collective distribution differences with or without alleles among the three most predictive QTL of (B) β‐glucan content, (D) protein content, and (F) oil content. The significance of differences between the groups was determined with Tukey's test (*α* = 0.05).

Four significant molecular markers across four chromosomes were identified for β‐glucan content in this study. Chromosomes 3A, 4C, 6D, and 7D each contained one SNP (Table S6). Significance values ranged from 1.3 to 2.3 (−log_10_ Hochberg & Benjamini *P* value, HBP), with the percent variation explained (PVE) by these markers ranging from 1.7% to 28.7% when the allele call was used as the estimator. Out of the four significant markers that met the initial PVE > 3 criteria, one additional marker was dropped due to a lack of durability across the three environments tested. Stepwise regression identified the three most predictive QTL from this dataset as Qβglucan.fgl‐6D, Qβglucan.fgl‐4C, and Qβglucan.fgl‐3A. Grouping the lines by the presence or absence of each QTL revealed significant differences in the normalized BLUE values (Table 3; Figure 2A) that were also observed across multiple environments (Table S7; Figure S5A–C). Qβglucan.fgl‐6D, the most predictive QTL, explained 4.2% of the variation for β‐glucan with presence of the QTL increasing β‐glucan content by approximately 0.6%. Further combining the three individual QTL into one haplotype explained 10.9% of the variation, and the presence of all three favorable QTL increased the β‐glucan content by 1.1% (Figure 2A,B). To be labeled as heart‐healthy by the FDA, oat varieties must consistently display a β‐glucan content of at least 4.5%. Being able to predict a 1% increase with markers alone will assist in early triage of poorly performing varieties and accelerate genetic gain for the trait.

The major QTL found here on 4C, tagged with S4C_27965926 (Table S6), was found on a large linkage block spanning approximately 2–60 Mb. Within this region, we identified previously reported CELLULOSE SYNTHASE‐LIKE 9 (CslF9_chr4C_24 Mb), QTL QBG.CORE‐BG_11.1, as well as Qbgl.UFRGS.L17N‐Mrg11.1 (Bazzer et al., 2025; Fogarty et al., 2019; Tinker et al., 2022). We also identified a *CslA7* gene (AVESA.00001b.r3.4Cg0000625 @ 47 Mb) that may also be implicated in this trait (Hazen et al., 2002). The cellulose synthase‐like gene family is known to be responsible for the hemicelluloses backbone synthesis of plant cell walls and β‐glucan synthesis in oat (Burton et al., 2006; Yin et al., 2009). Another QTL, Qβglucan.fgl‐6D, aligned to 6D:280 Mb on the Sang reference genome and is likely within the linkage block of the CslF9/11_Chr6D_254 Mb, another QTL/gene previously implicated with this trait (Tinker et al., 2022). Six other QTL on chromosome 7D were previously discovered and may be connected to the S7D_442623148 marker identified in this study that was removed upon filtering due to lack of durability across environments (Bazzer et al., 2025; Fogarty et al., 2019; Tinker et al., 2022). The mapping populations from which these β‐glucan QTL were identified involve the high‐β‐glucan parent, HiFi, which was developed by the NDSU program and is in the pedigree of many of the lines used in this study (McMullen et al., 2005).

### 3.5 QTL for protein of hexaploid oat

Five molecular markers located across five chromosomes (2A, 3C, 5A, 6A, and 7D) were identified as associated with protein content (Table S6). Significance values ranged from 1.8 to 8.9 (−log_10_ HBP) and PVE ranged from 4% to 10.6%. Two markers were removed from analysis due to lack of durable predictive ability in the three environments tested, and stepwise regression identified the three most predictive QTL as QProtein.fgl‐2A, QProtein.fgl‐5A, and QProtein.fgl‐3C. (Table 3; Figure 2C; Table S7; Figure S5D–F). Linkage analysis suggested that these QTL were present on co‐inherited blocks with physical sizes of 1.3, 96, and 516 Mb, respectively. QProtein.fgl‐2A was the most predictive QTL, explaining 6.1% of variation in protein, with presence of this QTL providing an approximate increase of between 0.6% and 0.9% in protein content over its absence (Table 3). Combining the three individual QTL into haplotypes explained 13.9% of variation (Figure 2D) with the presence of all three QTL in the haplotype predicting an approximate increase of 2%–3% over the absence of the haplotype (Table 3; Figure 2D).

The major QTL identified here, QProtein.fgl‐2A (S2A_398107713), was positioned ∼30 Mb proximal to a QTL identified by De Koeyer et al. (2004) and flanked by markers ISU707A/UMN464. Additionally, QProtein.fgl‐3C overlapped another reported grain protein content QTL on the long arm of 3C reported by Yan et al. (2023); however, the LD in our population was very high on chromosome 3C, so any connection between the two QTL may be coincidental. The last QTL selected in this study, QProtein.fgl‐5A, appears to be novel in relation to the handful of previously published mapping papers for grain protein content (Yan et al., 2023). With the high potential for elevated protein content in oat, relative to other small grains, further validation of these markers in more environments will be a priority for future studies.

### 3.6 QTL for oil of hexaploid oat

Groat oil percentage was found to be associated with six significant molecular markers on four chromosomes that explained 4.3%–15.7% of the observed variation (Table S6). LD analysis of the genotyping data suggested no linkage between the three markers identified on chromosome 6A. Stepwise regression analysis indicated the three most predictive QTL to be QOil.fgl‐4C, QOil.fgl‐6A, and QOil.fgl‐5A, explaining 16.9%, 11%, and 7.3% of variation, respectively (Table 3; Figure 2E; Table S7; Figure S5G–I).

The QTL QOil.fgl‐4C displayed a slightly greater PVE (16.9% vs. 15.9%) when the marker was evaluated as a binary (±) versus the original genotype call with three allele states. We estimated an approximate increase of 0.6% in oil content when the QTL was present. Additionally, the presence of all three QTL in a haplotype predicted an increase of approximately 1% oil content over the absence of the haplotype in this population (Figure 2A).

Among the QTL identified here for oil content in the NDSU oat breeding program (Table S2), those on chromosomes 4C and 6A were nearby five markers/QTL identified in previous studies (Campbell et al., 2021; Kianian et al., 1999; Orr & Molnar, 2007; Tinker et al., 2022; Zhu et al., 2004). One of these markers, S4C_21549747 (QOil.fgl‐4C), was located within 100 kb of marker CDO665_WHE03A39.r1 and the linked ACCaseA locus, which has previously been found associated with groat oil content in several biparental mapping populations—Kanota × Ogle, Kanota × Marion, Dal × Exeter, and Goslin × Hifi (Kianian et al., 1999; Tinker et al., 2022).

The genome regions underlying QOil.fgl‐4C, QOil.fgl‐6A, and QOil.fgl‐5A contained 821, 30, and 13 annotated genes, respectively (Table S8). The gene *AVESA.00001b.r3.4Cg0000200* located at 15.1 Mb was within the LD block of our identified QTL and shares protein homology (91% identity) to the previously reported ACCase gene (Kianian et al., 1999; Tinker et al., 2022). A duplicated ACCase gene (*AVESA.00001b.r3.6Ag0003033* at 420.6 Mb) is positioned nearby (∼3 Mb) QOil.fgl‐6A. ACCase catalyzes the formation of ATP‐dependent malonyl‐CoA from acetyl‐CoA and bicarbonate (Harwood, 1988) and plays an essential role in the initial stage of fatty acid biosynthesis in oat. These two genes are likely strong influencers of oil in the oat grain, and natural variation at these loci may uncover new ways to manipulate the quantity and quality of this important nutrient.

### 3.7 QTL for test weight of hexaploid oat

**FIGURE 3:**
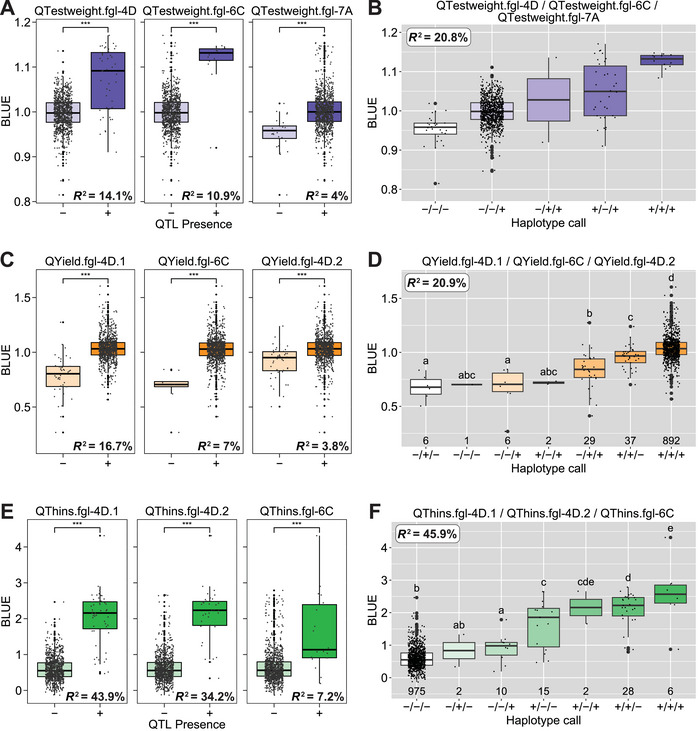
Distributions of three most useful quantitative trait loci (QTL) indicate significant differences using best linear unbiased estimator (BLUE) value with or without alleles of the specific markers of (A) test weight, (C) yield, (E) thins. The significance of differences between the pairwise means was determined with the Wilcox test (***p ≤ 0.001). Haplotype calls of collective distribution differences with or without alleles among the three most predictive QTL of (B) test weight, (D) yield, (F) thins. The significance of differences between the groups was determined with Tukey's test (*α* = 0.05).

Nine significant markers were identified for test weight, on chromosomes 1D, 2C, 2D, 6C, 7A, 7C, and 7D (Table S6). The significance values ranged from 1.8 to 12.2 (−log_10_ HBP), and the variation explained by these markers ranged from 3.4% to 13.1%. Three of the nine markers were removed because they were not durably predictive across the 10 environments tested. Stepwise regression identified the three most useful markers, QTestweight.fgl‐4D, QTestweight.fgl‐6C, and QTestweight.fgl‐7A, that explained 14.1%, 10.9%, and 4.0% of variation, respectively (Table 3; Figure 3A; Table S7; Figure S6A–C). Significant differences in the trait BLUE values were observed when lines were grouped by the presence or absence of a given QTL. The presence of the most predictive QTL, QTestweight.fgl‐4D, provided an approximate 4.5 kg/hL increase in test weight over its absence. A combination of the three individual QTL into haplotypes explained 20.8% of variation (Table 3; Figure 3B), and an average test weight increase of 7.5–9 kg/hL when all three favorable QTL are present vs. absent within a line (Figure 3A).

Several test weight QTL have been detected from recombinant inbred populations in the literature (De Koeyer et al., 2004; Tinker et al., 2022) with the most important test weight QTL being the N1 locus at 411 Mb (Sang reference; Kamal et al., 2022) that confers the covered phenotype to the oat grain (Wang et al., 2023 and references therein). In this study, QTestweight.fgl‐4D aligned to 415 Mb on the Sang reference sequence and is also likely the N1 locus. This is interesting as the naked trials only represented 5% of the total plots tested in this study, validating the large effect of the loci. The other two QTL identified as most useful in this study appear to be novel; however, the high environmental impact on this trait suggests that several more years of data will be necessary to make an inference about a causative mechanism.

### 3.8 QTL for groat of hexaploid oat

Four significant markers were identified for groat percentage, with two markers each located on chromosomes 4D and 7D (Table S6). Evaluation of the LD heatmaps suggested linkage block sizes of ∼10 Mb, so the four markers represented unique QTL. The variation explained by each identified SNP ranged from 4.3% to 9.4%, and marker significance values ranged from 2 to 5.7 (−log_10_ HBP). Evaluation of the PVE within each environment showed that only a single marker (S4D_361348095) was durably predictive in the three locations it was measured in (Table S7). This QTL, QGroat.fgl‐4D, explained 7.1% of variation in groat with its presence predicting a 3% increase in groat content overall and within individual environments (Table S7; Figure S6D). It does not appear to overlap the N1 locus (∼90 Mb distal) or any of the other previously reported milling QTL (Esvelt Klos et al., 2021; Tinker et al., 2022).

### 3.9 QTL for yield of hexaploid oat

Seven significant markers were identified for yield, with a single SNP located on chromosomes 2D, 3C, 4C, 6C, and 7D (Table S6). Two markers each were identified on chromosome 4D. Significance values ranged from 3.1 to 20.9 (−log_10_ HBP), and the variation explained by these QTL ranged from 1.2% to 43.4% (Table S6). Before the stepwise regression analysis to determine the three most predictive QTL, three of the significant markers were excluded because they were not durably predictive in the 11 tested environments. QYield.fgl‐4D.1, QYield.fgl‐6C, and QYield.fgl‐4D.2 were the most predictive QTL, explaining 16.7%, 7.0%, and 3.8% of variation, respectively (Table 3; Figure 3C; Table S7; Figure S6G–I). The most predictive QTL, QYield.fgl‐4D.1, provides an average yield increase of between 600 and 1500 kg/ha, depending on the environment (Figure S6I). When all three were collectively grouped into haplotypes, 20.9% of the variation was explained by the markers with a predicted increase of 800–2000 kg/ha in yield when the three QTL are present versus absent (Table 3; Figure 3D).

The most informative yield QTL identified here, QYield.fgl‐4D.1, resides on the same linkage block as the QTestweight.fgl‐4D and the presumed N1 locus. Along with the covered (hulless, naked) phenotype, the loci determines several additional traits such as thins (%), 1000‐kernel weight, groat content, and yield (Tinker et al., 2022). With such dramatic effects, it is possible that the causative agent underlying the N1 locus is a transcription regulator, of which four genes were identified within the QYield.fgl‐4D.1 linkage block that contain this function (AVESA.00001b.r3.4Dg000—3913, 3921, 3930, and 3935; Table S8).

### 3.10 QTL for thins of hexaploid oat

The thins metric represents the portion of kernels that fall below a size threshold determined for market desirability. In this study, seven significant molecular markers for thins were identified on four chromosomes, with three and two markers located on chromosomes 4D and 6C, respectively. A single SNP each was identified on chromosomes 3A and 4C (Table S6). LD analysis suggested no linkage between the three markers on 4D and the two markers on 6C, indicating the presence of distinct QTL. Significance values ranged from 3.1 to 43.0 (−log_10_ HBP, Table S6). QThins.fgl‐1D.1, QThins.fgl‐4D.2, and QThins.fgl‐6C were the three most predictive QTL identified by stepwise regression, explaining 43.9%, 34.2%, and 7.2% of variation, respectively (Table 3; Figure 3E; Table S7; Figure S6J–L). Three significant markers located on 3A, 4D, and 6C were removed before the stepwise regression analysis since they were not durable across the 11 environments. The most predictive QTL, QThins.fgl‐4D.1, provides an approximate average increase of 28%–50% over the absence. When grouped as a haplotype, the QTL explained a similar amount of variation (45.9%) as QThins.fgl‐4D.1 (Figure 3F), but predicted a larger average increase of 40%–70%% over the absence of the haplotype, suggesting that breeding efforts aiming to increase marketable yield should select for lines that do not contain these QTL. Again, the major QTL found for this trait appears to overlap the N1 locus described above, and the second most useful QTL, QThins.fgl‐4D.2, was very close by. The influence of many traits by this single locus is well characterized (Wang et al., 2023) and is expected by the significant correlation among thins, yield, and test weight traits in this study (Figure 1).

### 3.11 QTL for heading date of hexaploid oat

**FIGURE 4:**
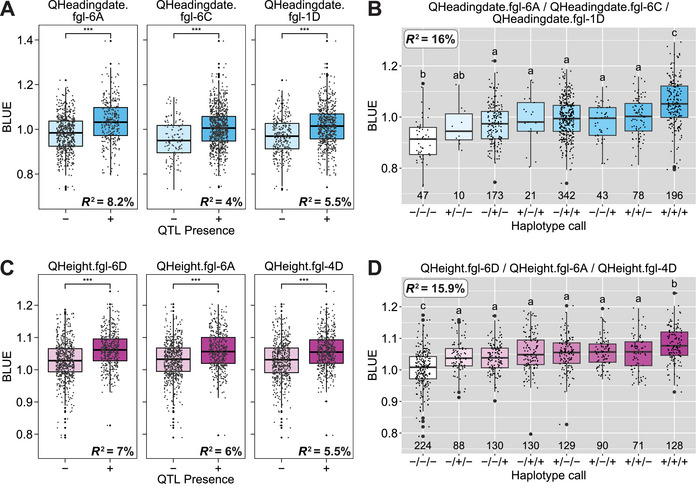
Distributions of three most useful quantitative trait loci (QTL) indicate significant differences using best linear unbiased estimator (BLUE) value with or without alleles of the specific markers of (A) heading date and (C) height. The significance of differences between the pairwise means was determined with the Wilcox test (***p ≤ 0.001). Haplotype calls of collective distribution differences with or without alleles among the three most predictive QTL of (B) heading date and (D) height. The significance of differences between the groups was determined with Tukey's test (*α* = 0.05).

Eighteen significant markers were identified for heading date across 11 chromosomes. Chromosomes 1D, 2A, 3C, 4D, 5A, 5C, and 6C each contained a single marker, while chromosomes 4A, 5A, and 6A each contained two markers, and five markers were identified on 7D (Table S6). Significant values ranged from 1.4 to 12 (−log_10_ HBP), and the variation explained by these markers ranged from 3.0% to 11.6%. QHeadingdate.fgl‐6A, QHeadingdate.fgl‐6C, and QHeadingdate.fgl‐1D were identified by stepwise regression as the most predictive QTL, explaining 8.2%, 4.0%, and 5.5% of variation, respectively, after five markers were removed due to lack of durability across environments (Table 3; Figure 4A; Table S7; Figure S7A–C). Significant differences in the trait BLUE values were identified when lines were grouped by the presence or absence of a specified QTL. Presence of the most predictive QTL, QHeadingdate.fgl‐6A, increased the days until heading by approximately 5–6 days. Combining the three individual QTL into haplotypes explained 16% of the variation (Table 3), and presence of all three QTL in a haplotype predicted an average increase of 12–16 days to heading over the absence of the haplotype (Table 3; Figure 4B), depending on the environment (Figure S7A–C).

Heading date has been the focus of several QTL mapping studies. Zimmer et al. (2018) identified genomic regions controlling heading date in two hulled by naked oat populations. One significant SNP marker, GMI_ES15_c15940_121, aligned to 6A:419 Mb, very close to the linkage block surrounding QHeadingdate.fgl‐6A. A recent report characterized several heading date–related QTL using two biparental mapping populations (de Ubert & Nava, 2024) and identified a QTL on 6A (Mrg05) that overlaps with QHeadingdate.fgl‐6A. Underlying this QTL, two annotated genes contain flowering time functions–the PSEUDO RESPONSE REGULATOR‐like (PRR37, AVESA.00001b.r3.6Ag0002826 @ 6A:405.7 Mb) and a Putative F‐box/FBD/LRR‐repeat protein (FDL24, AVESA.00001b.r3.6Ag0002728 @ 6A:397 Mb)–and are likely playing influential roles in this population. Another heading date QTL discovered in this study, QHeadingdate.fgl‐1D, is also nearby several QTL found in the literature (Canales et al., 2021; de Ubert & Nava, 2024; Siripoonwiwat et al., 1996) with the FRIGIDA‐like 3 (FRL3, AVESA.00001b.r3.1Dg0001847 @ 1D:347.6 Mb) gene as a potential causative agent.

### 3.12 QTL for height of hexaploid oat

Five markers were identified for height across chromosomes 4D, 6A, and 6C (Table S6). Chromosome 6D had two markers identified on it, and LD analysis of the genotyping data suggested no linkage among those two molecular markers. Significance values of all identified markers ranged from 1.3 to 2.3 (−log_10_ HBP), and the PVE ranged from 4.2% to 7.0%. All the significant markers were found to be durably predictive in the six environments tested. The three most predictive QTL identified by stepwise regression from this data set were named QHeight.fgl‐6D, QHeight.fgl‐6A, and QHeight.fgl‐4D, explaining 7%, 6%, and 5.5% of variation, respectively (Table 3; Figure 4C; Table S7; Figure S7D–F). All three QTL exhibited the same effect, with the presence of QTL, increasing the height of the line approximately 2 cm over its absence (Table 3). Combining the three most predictive QTL into haplotypes resulted in a PVE of 15.9% (Figure 4D). Shorter plants are usually desirable in high‐production regions to limit lodging at the end of the growing season; thus, breeders selecting for the lines without these three QTL should expect an average decrease in height of approximately 6 cm.

The QTL identified in this study (QHeight.fgl‐6D) located on 6D from 278 to 291 Mb are close to several other previously published QTL for height. Zimmer et al. (2018) identified a plant height QTL flanked by markers GMI_ES02_c1838_173 and GMI_GBS_13021 that align to 6D:222 and 6D:230 Mb, respectively. These QTL may be linked to the dwarfing gene, *Dw‐6*, mapped to chromosome 6D:237 Mb (Yan et al., 2021). A recent publication also identified several plant height QTL from a Dal/Exeter mapping population that are associated with marker avgbs_13292.1 and contain the 244–270 Mb region of Sang chromosome 6D (Tinker et al., 2022).

## 4 CONCLUSION

The simultaneous improvement of many different crop characteristics is a laborious and time‐consuming task for small grains breeding programs. Due to these difficulties, many traits necessary for cultivar development are not directly evaluated until germplasm is well‐advanced and evaluated in multiple field locations. When direct phenotyping cannot take place, early genotyping with predictive molecular markers can enhance breeding efficiency by enabling the early triage of lines predicted to possess undesirable traits. Depending on the molecular breeding scheme employed, larger pools of early‐generation material can be assessed. More stringent selection criteria can also be used, or both methods can be employed to increase the speed and efficiency of bringing new cultivars to the market. The durable predictive markers we have identified in this study will be useful for facilitating these goals in oat breeding programs. Identification of genes located near the QTL for each studied trait also has provided a starting point for future functional molecular studies.

## AUTHOR CONTRIBUTIONS

**Naa Korkoi Ardayfio**: Data curation; formal analysis; investigation; methodology; project administration; writing—original draft; writing—review and editing. **Christy Kinney**: Visualization; writing—original draft; writing—review and editing. **Dandan Li**: Investigation; writing—original draft; writing—review and editing. **Sepehr Mohajeri Naraghi**: Data curation; methodology; resources; writing—review and editing. **Michael S. McMullen**: Methodology; project administration; resources; writing—review and editing. **Jason D. Fiedler**: Conceptualization; data curation; funding acquisition; investigation; project administration; supervision; writing—review and editing.

## CONFLICT OF INTEREST STATEMENT

The authors declare no conflicts of interest.

## Supporting information




**Supplementary Table 1** contains PYT/AYT specific and unique genotype number with traits measured, locations and replications across years.
**Supplementary Table 2** contains trait data used in this study.
**Supplementary Table 3** contains repeated genotypes across experimental trials and years.
**Supplementary Table 4** contains spatially adjusted means for each environment.
**Supplementary Table 5** contains normalized BLUE values (check values excluded prior to association analysis).
**Supplementary Table 6** contains relevant information for all markers identified with GWAS.
**Supplementary Table 7** contains Significant markers per‐environment PVE markers before stepwise regression analysis.
**Supplementary Table 8** Annotated genes within QTL linkage blocks.

**Supplementary Figure 1** contains the distribution of spatially adjusted environment means for β‐Glucan (A), Protein (B), Oil (C), Test weight (D), Groat (E), Yield (F), Thins (G), Heading date (H) and Height (I).

**Supplementary Figure 2** contains the principal component analysis of the taxa used in this study.

**Supplementary Figure 3** contains the GWAS results visualized with QQ and Manhattan plots for β‐Glucan (A,B), Protein (C,D), Oil (E,F), Test weight (G,H), Groat (I,J), Yield (K,L), Thins (M,N), Heading date (O,P) and Height (Q,R).

**Supplementary Figure 4** contains the distribution of normalized BLUE values grouped by allele state.

**Supplementary Figure 5** contains the distribution of spatially adjusted environment means grouped by the three most significant QTL for β‐Glucan (A‐C), Protein (D‐F), and Oil (G‐I).

**Supplementary Figure 6** contains the distribution of spatially adjusted environment means grouped by the three most significant QTL for Test weight (A‐C), Groat (D‐F), Yield (G‐I), and Thins (J‐L).

**Supplementary Figure 7** contains the distribution of spatially adjusted environment means grouped by the three most significant QTL for Heading Date (A‐C) and Height (D‐F).

## Data Availability

Genotyping data have been deposited in the T3/Oat database under the “NDSU GBS” genotyping protocol. Phenotyping data can be found in Table S2.
