# Supplementary material for: Genome‐wide association studies reveal genetic control of nutritional quality, milling traits, and agronomic characteristics in oat (Avena sativa L.)
Source: Plant Genome. 2025 Jul 7;18(3):e70060. doi: 10.1002/tpg2.70060 (PMC12234416; doi:10.1002/tpg2.70060)
Supplement: Supplementary file 1 [file TPG2-18-e70060-s002.pdf]

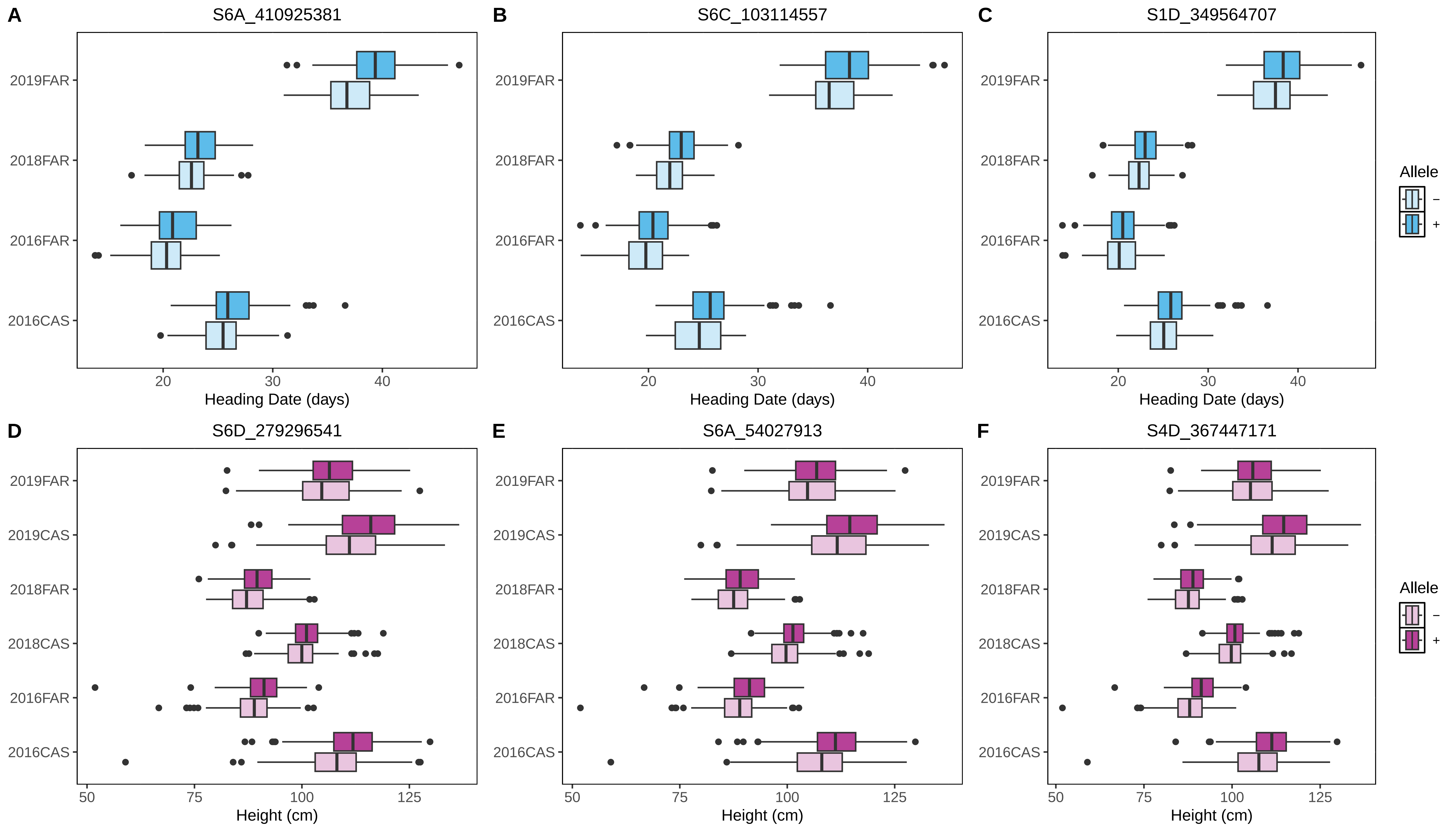

A
S6A_410925381
B
S6C_103114557
C
S1D_349564707
2019FAR
2018FAR
2016FAR
2016CAS
20
30
40
Heading Date (days)
Allele
–
+
D
S6D_279296541
E
S6A_54027913
F
S4D_367447171
2019FAR
2019CAS
2018FAR
2018CAS
2016FAR
2016CAS
50
75
100
125
Height (cm)
