# Supplementary material for: Genome‐wide association studies reveal genetic control of nutritional quality, milling traits, and agronomic characteristics in oat (Avena sativa L.)
Source: Plant Genome. 2025 Jul 7;18(3):e70060. doi: 10.1002/tpg2.70060 (PMC12234416; doi:10.1002/tpg2.70060)
Supplement: Supplementary file 2 [file TPG2-18-e70060-s003.pdf]

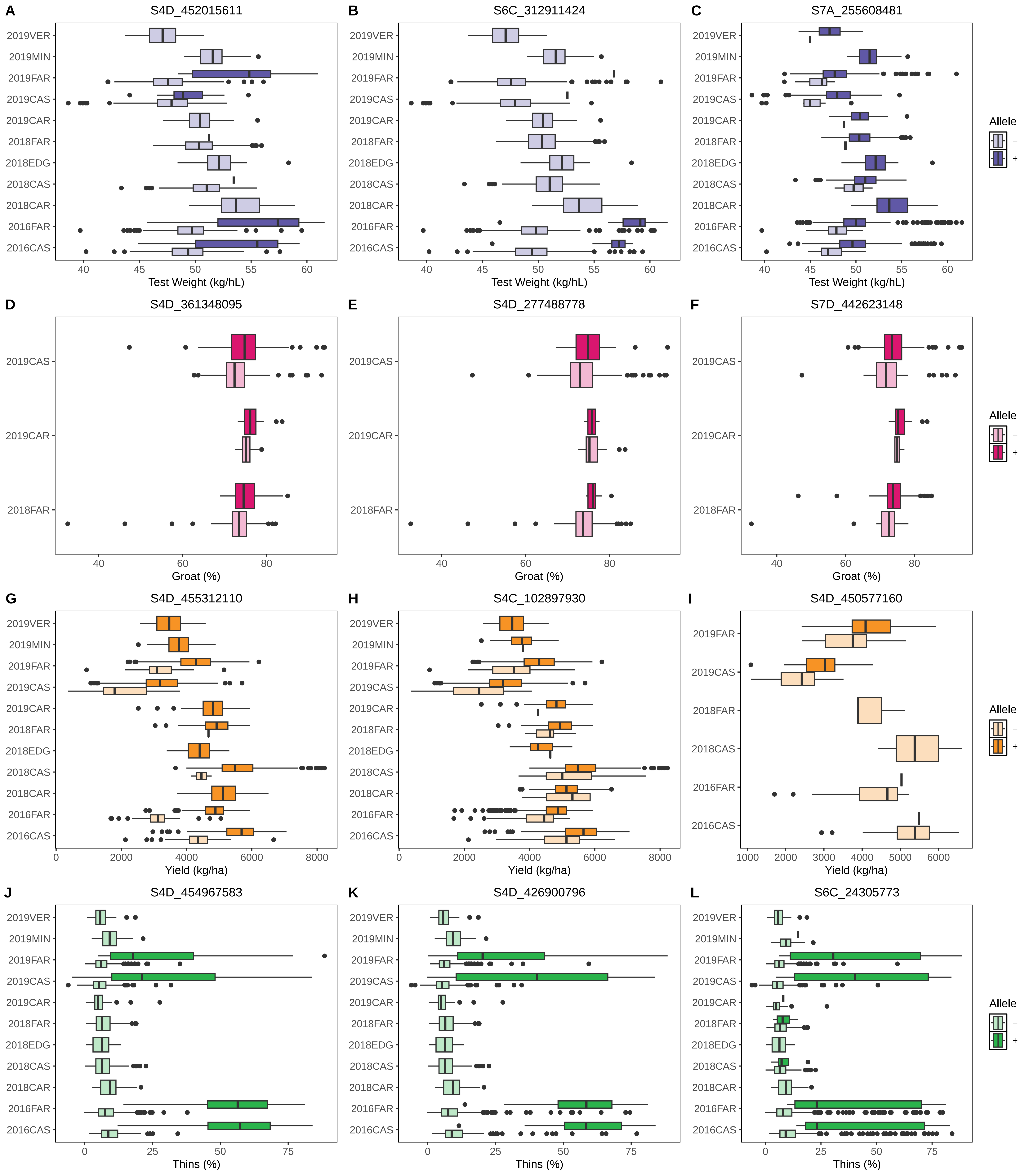

A
S4D_452015611
B
S6C_312911424
C
S7A_255608481
2019VER
2019MIN
2019FAR
2019CAS
2019CAR
2018FAR
2018EDG
2018CAS
2018CAR
2016FAR
2016CAS
Test Weight (kg/hL)
Allele
−
+
D
S4D_361348095
E
S4D_277488778
F
S7D_442623148
Groat (%)
G
S4D_455312110
H
S4C_102897930
I
S4D_450577160
Yield (kg/ha)
J
S4D_454967583
K
S4D_426900796
L
S6C_24305773
Thins (%)
