# Supplementary material for: Genome‐wide association studies reveal genetic control of nutritional quality, milling traits, and agronomic characteristics in oat (Avena sativa L.)
Source: Plant Genome. 2025 Jul 7;18(3):e70060. doi: 10.1002/tpg2.70060 (PMC12234416; doi:10.1002/tpg2.70060)
Supplement: Supplementary file 3 [file TPG2-18-e70060-s004.pdf]

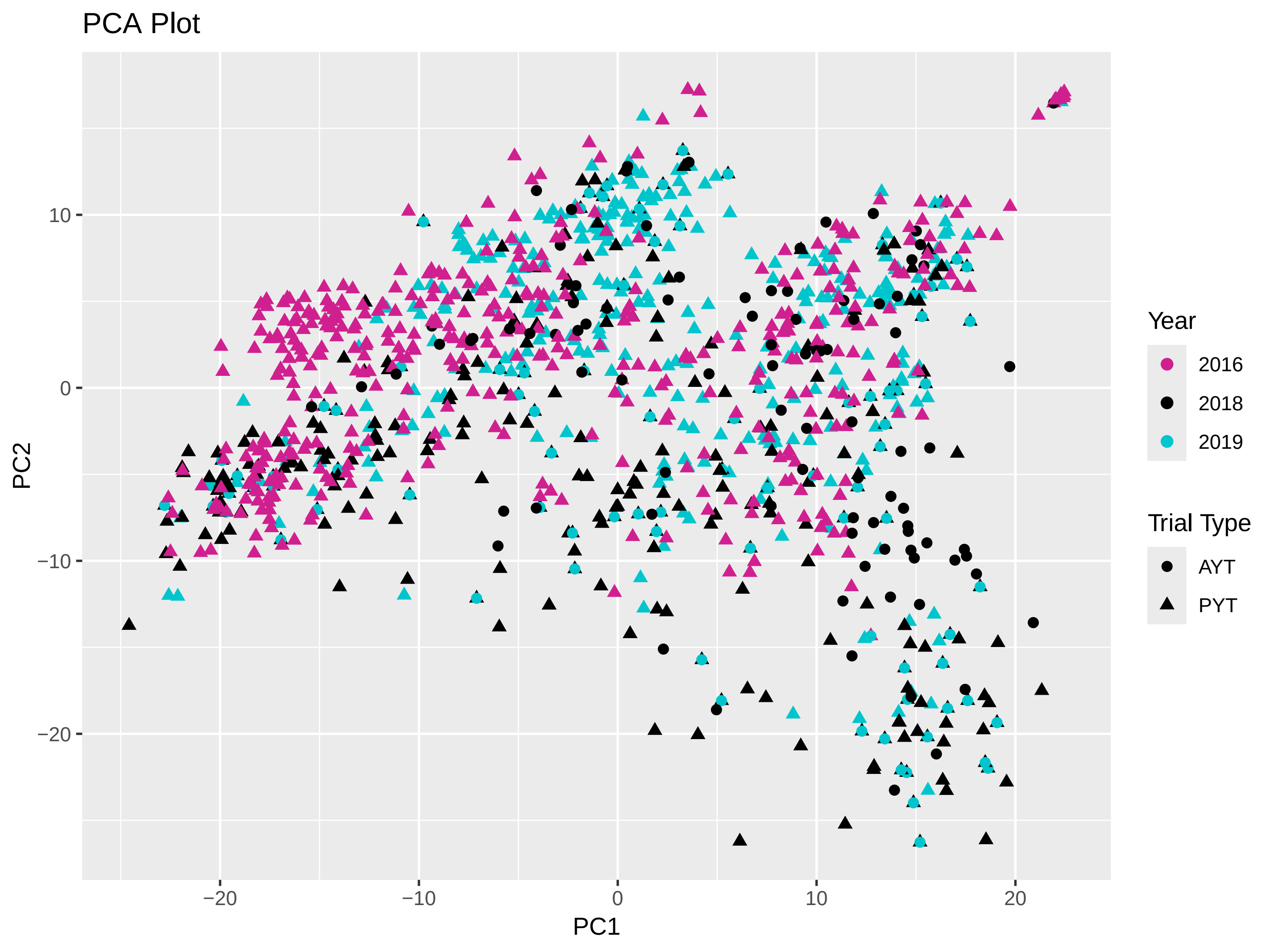
PCA Plot
PC2
10
0
−10
−20
−20
−10
0
10
20
PC1
Year
2016
2018
2019
Trial Type
AYT
PYT
