# Supplementary material for: Genome‐wide association studies reveal genetic control of nutritional quality, milling traits, and agronomic characteristics in oat (Avena sativa L.)
Source: Plant Genome. 2025 Jul 7;18(3):e70060. doi: 10.1002/tpg2.70060 (PMC12234416; doi:10.1002/tpg2.70060)
Supplement: Supplementary file 4 [file TPG2-18-e70060-s005.pdf]

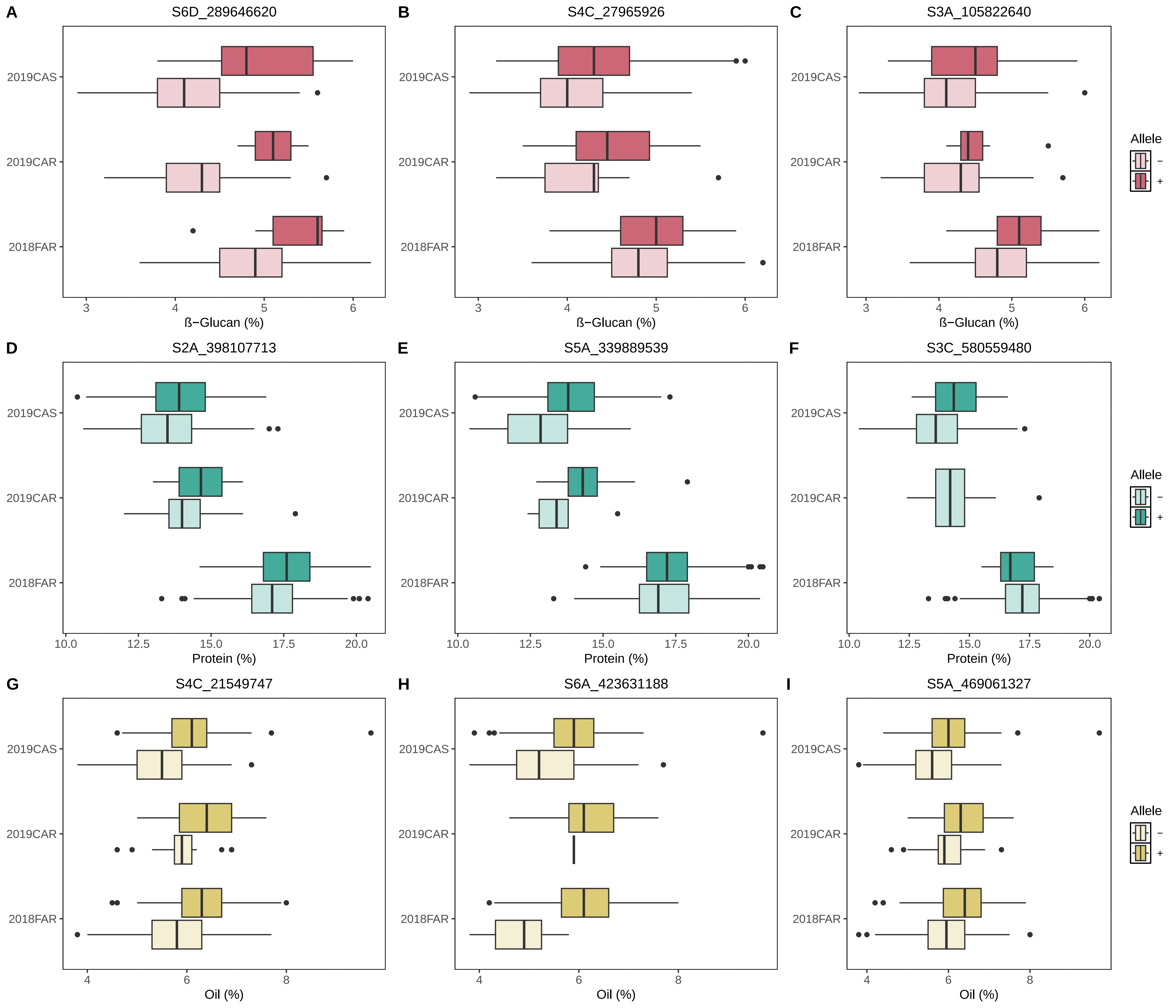
A
S6D_289646620
B
S4C_27965926
C
S3A_105822640
2019CAS
2019CAR
2018FAR
ß−Glucan (%)
Allele
−
+
D
S2A_398107713
E
S5A_339889539
F
S3C_580559480
Protein (%)
G
S4C_21549747
H
S6A_423631188
I
S5A_469061327
Oil (%)
