# Supplementary material for: Genome‐wide association studies reveal genetic control of nutritional quality, milling traits, and agronomic characteristics in oat (Avena sativa L.)
Source: Plant Genome. 2025 Jul 7;18(3):e70060. doi: 10.1002/tpg2.70060 (PMC12234416; doi:10.1002/tpg2.70060)
Supplement: Supplementary file 5 [file TPG2-18-e70060-s006.pdf]

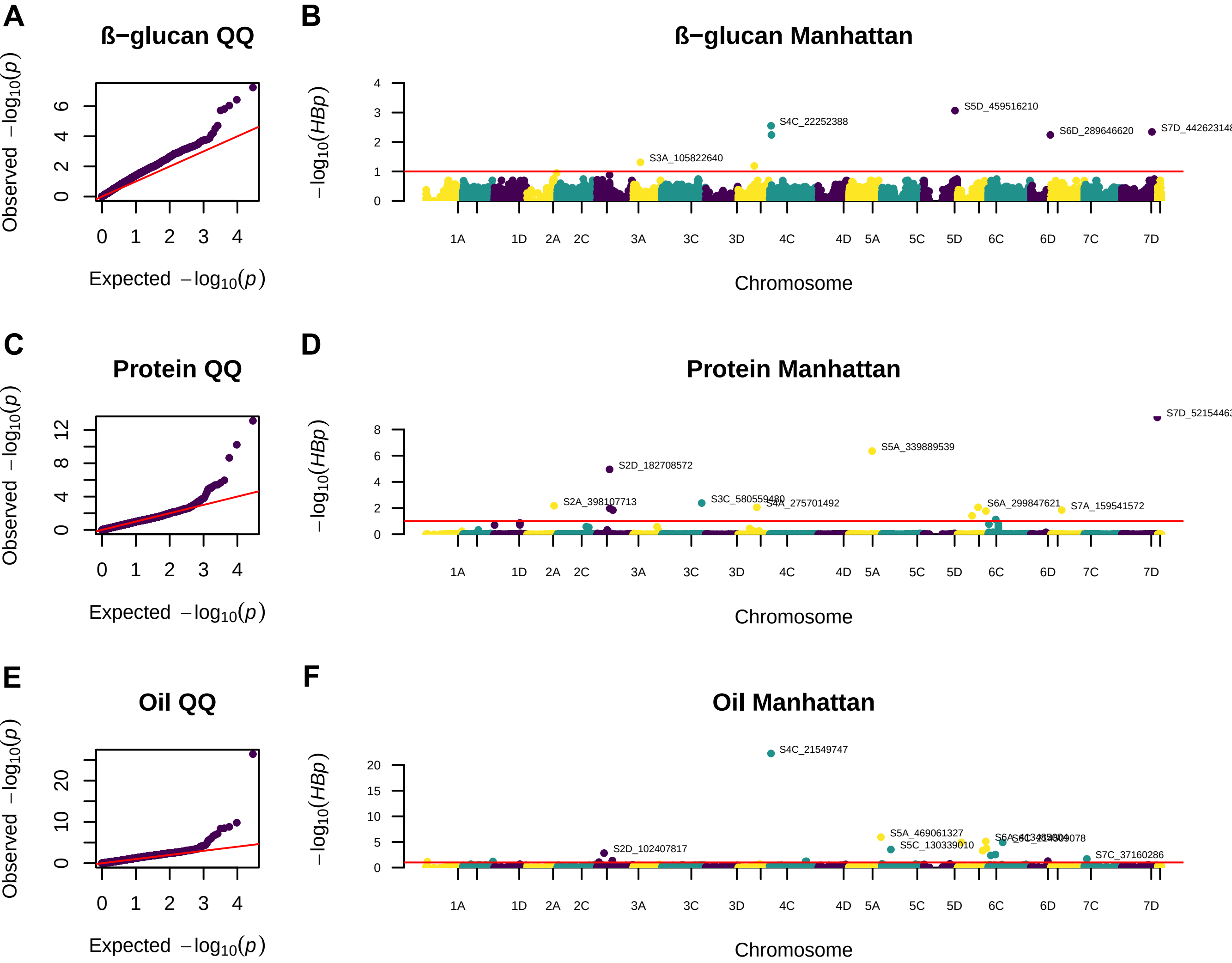

A
ß−glucan QQ
Observed −log10(p)
Expected −log10(p)
B
ß−glucan Manhattan
−log10(HBp)
S5D_459516210
S4C_22252388
S6D_289646620
S3A_105822640
Chromosome
1A
1D
2A
2C
3A
3C
3D
4C
4D
5A
5C
5D
6C
6D
7C
7D
C
Protein QQ
D
Protein Manhattan
S5A_339889539
S2D_182708572
S2A_398107713
S6A_299847621
S7A_159541572
E
Oil QQ
F
Oil Manhattan
S4C_21549747
S5A_469061327
S5C_130339010
S2D_102407817
S7C_37160286

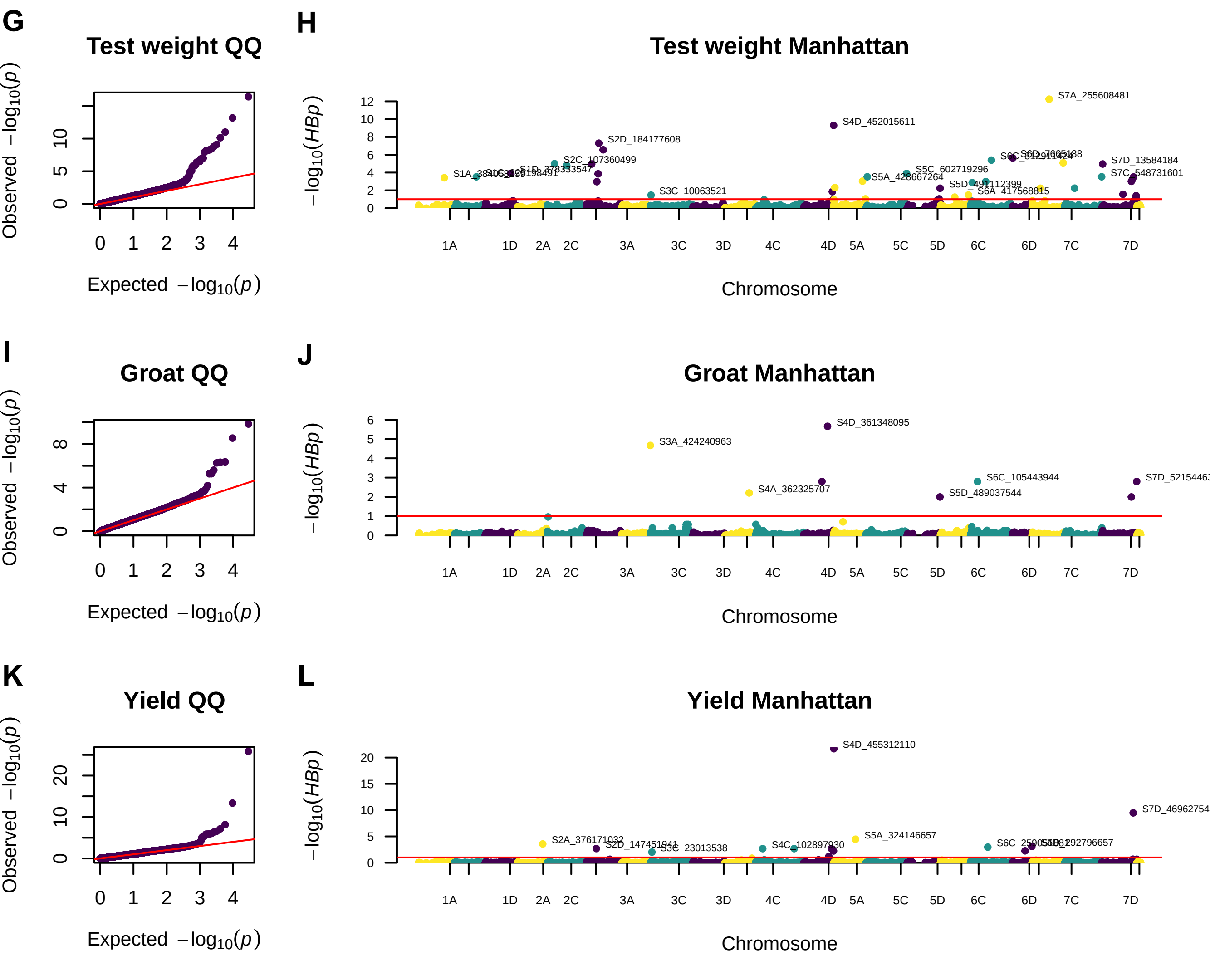
G
Test weight QQ
Observed −log10(p)
Expected −log10(p)
H
Test weight Manhattan
−log10(HBp)
S7A_255608481
S4D_452015611
S2D_184177608
S2C_107360499
S7D_13584184
S7C_548731601
S5C_602719296
S5A_428667264
S3C_10063521
S6A_417568815
Chromosome
1A 1D 2A 2C 3A 3C 3D 4C 4D 5A 5C 5D 6C 6D 7C 7D
I
Groat QQ
Observed −log10(p)
Expected −log10(p)
J
Groat Manhattan
−log10(HBp)
S4D_361348095
S3A_424240963
S6C_105443944
S4A_362325707
S5D_489037544
Chromosome
1A 1D 2A 2C 3A 3C 3D 4C 4D 5A 5C 5D 6C 6D 7C 7D
K
Yield QQ
Observed −log10(p)
Expected −log10(p)
L
Yield Manhattan
−log10(HBp)
S4D_455312110
S5A_324146657
S3C_23013538
Chromosome
1A 1D 2A 2C 3A 3C 3D 4C 4D 5A 5C 5D 6C 6D 7C 7D

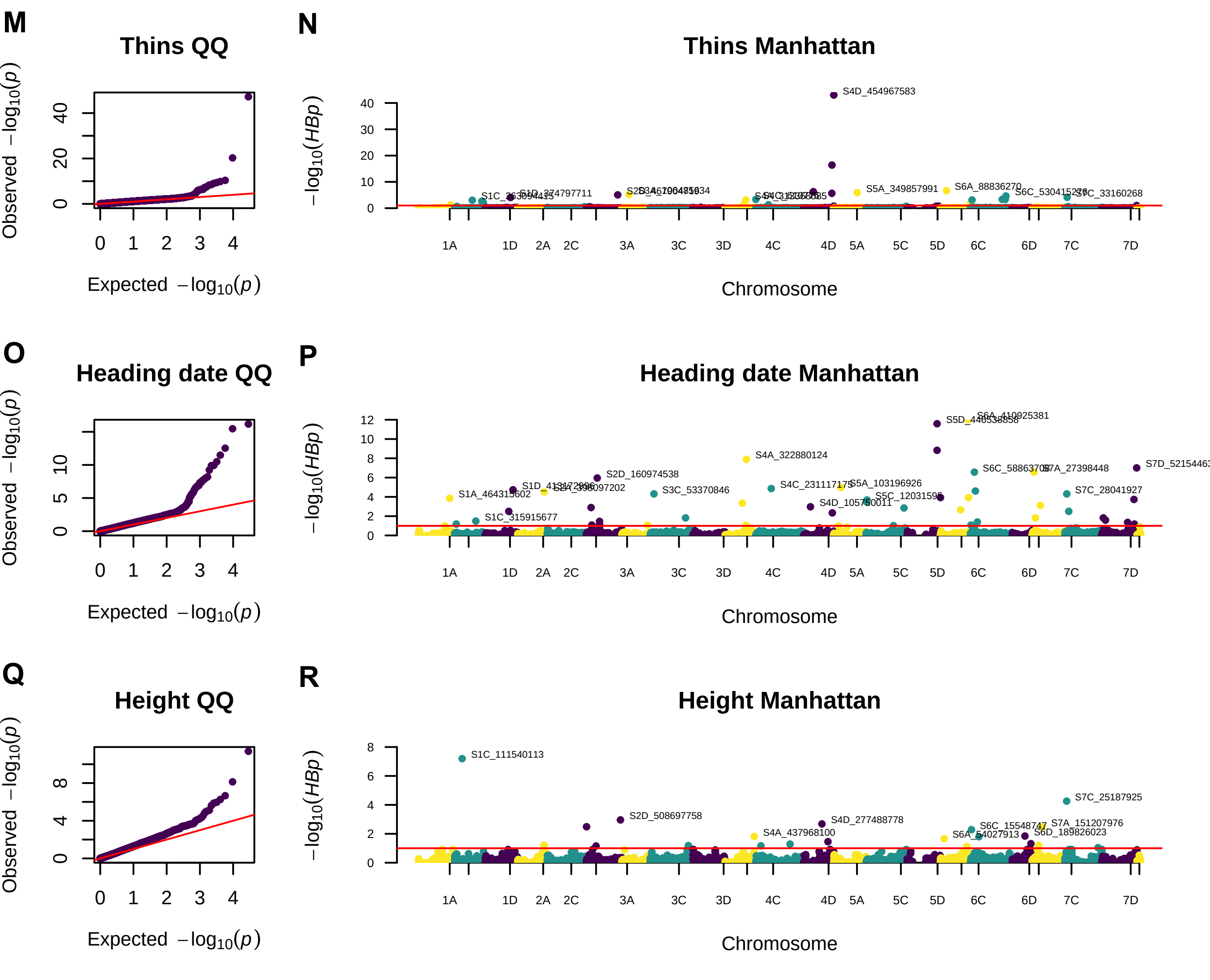

M
Thins QQ
Observed −log10(p)
Expected −log10(p)
N
Thins Manhattan
−log10(HBp)
S4D_454967583
S1C_263094415
S1D_374797711
S5A_349857991
S6A_88836270
S7C_33160268
Chromosome
O
Heading date QQ
Observed −log10(p)
Expected −log10(p)
P
Heading date Manhattan
−log10(HBp)
S6A_410925381
S4A_322880124
S2D_160974538
S7A_27398448
S1A_464315602
S1C_315915677
S3C_53370846
S5A_103196926
S5C_12031595
S7C_28041927
Chromosome
Q
Height QQ
Observed −log10(p)
Expected −log10(p)
R
Height Manhattan
−log10(HBp)
S1C_111540113
S7C_25187925
S2D_508697758
S4D_277488778
S4A_437968100
S6C_15548747
S7A_151207976
S6A_54027913
S6D_189826023
Chromosome
