# Supplementary material for: Genome‐wide association studies reveal genetic control of nutritional quality, milling traits, and agronomic characteristics in oat (Avena sativa L.)
Source: Plant Genome. 2025 Jul 7;18(3):e70060. doi: 10.1002/tpg2.70060 (PMC12234416; doi:10.1002/tpg2.70060)
Supplement: Supplementary file 6 [file TPG2-18-e70060-s007.pdf]

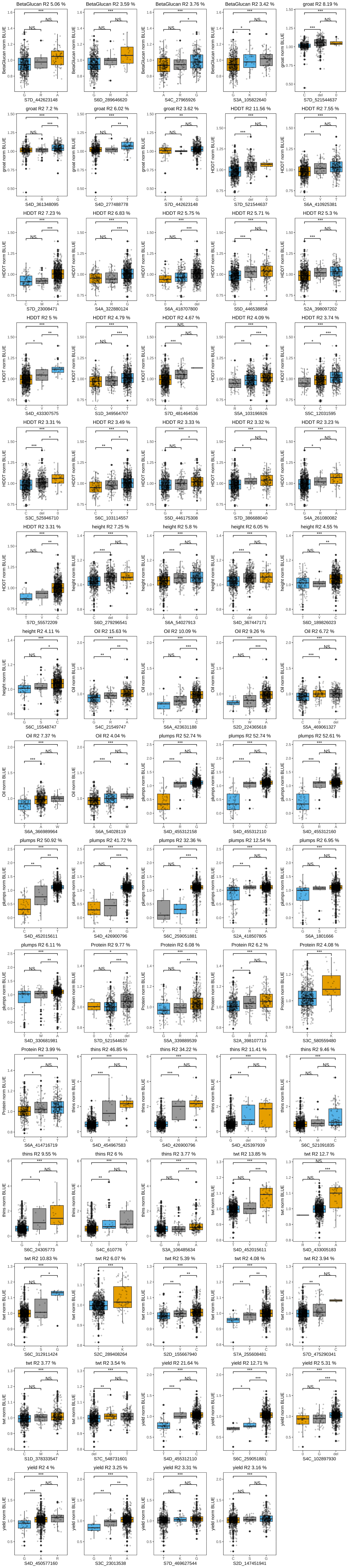
BetaGlucan R2 5.06 %
S7D_442623148
BetaGlucan R2 3.59 %
S6D_289646620
BetaGlucan R2 3.76 %
S4C_27965926
BetaGlucan R2 3.42 %
S3A_105822640
groat R2 8.19 %
S7D_521544637
groat R2 7.2 %
S4D_361348095
groat R2 6.02 %
S4D_277488778
groat R2 3.62 %
S7D_442623148
HDDT R2 11.56 %
S7D_521544637
HDDT R2 7.55 %
S6A_410925381
HDDT R2 7.23 %
S7D_23008471
HDDT R2 6.83 %
S4A_322880124
HDDT R2 5.75 %
S6A_418707800
HDDT R2 5.71 %
S5D_446538858
HDDT R2 5.3 %
S2A_398097202
HDDT R2 5 %
S4D_433307575
HDDT R2 4.79 %
S1D_349564707
HDDT R2 4.67 %
S7D_481464536
HDDT R2 4.09 %
S5A_103196926
HDDT R2 3.74 %
S5C_12031595
HDDT R2 3.31 %
S3C_525946710
HDDT R2 3.49 %
S6C_103114557
HDDT R2 3.33 %
S5D_446175308
HDDT R2 3.32 %
S7D_386688040
HDDT R2 3.23 %
S4A_261080082
HDDT R2 3.31 %
S7D_55572209
height R2 7.25 %
S6D_279296541
height R2 5.8 %
S6A_54027913
height R2 6.05 %
S4D_367447171
height R2 4.55 %
S6D_189826023
height R2 4.11 %
S6C_15548747
Oil R2 15.63 %
S4C_21549747
Oil R2 10.09 %
S6A_423631188
Oil R2 9.26 %
S2D_224365618
Oil R2 6.72 %
S5A_469061327
Oil R2 7.37 %
S6A_366989964
Oil R2 4.04 %
S6A_54028119
plumps R2 52.74 %
S4D_455312158
plumps R2 52.74 %
S4D_455312110
plumps R2 52.61 %
S4D_455312160
plumps R2 50.92 %
S4D_452015611
plumps R2 41.72 %
S4D_426900796
plumps R2 32.36 %
S6C_259051881
plumps R2 12.54 %
S2A_418507805
plumps R2 6.95 %
S6A_1801666
plumps R2 6.11 %
S4D_330681981
Protein R2 9.77 %
S7D_521544637
Protein R2 6.08 %
S5A_339889539
Protein R2 6.2 %
S2A_398107713
Protein R2 4.08 %
S3C_580559480
Protein R2 3.99 %
S6A_414716719
thins R2 46.85 %
S4D_454967583
thins R2 34.22 %
S4D_426900796
thins R2 11.41 %
S4D_425397939
thins R2 9.46 %
S6C_521091835
thins R2 9.55 %
S6C_24305773
thins R2 6 %
S4C_621776
thins R2 3.77 %
S3A_106485634
twt R2 13.85 %
S4D_452015611
twt R2 12.7 %
S4D_433005183
twt R2 10.83 %
S6C_312911424
twt R2 6.07 %
S2C_289408264
twt R2 5.39 %
S2D_155667940
twt R2 4.08 %
S7A_255608481
twt R2 3.94 %
S7D_475290341
twt R2 3.77 %
S1D_378333547
twt R2 3.54 %
S7C_548731601
yield R2 21.64 %
S4D_455312110
yield R2 12.71 %
S6C_259051881
yield R2 5.31 %
S4C_102897930
yield R2 4 %
S4D_450577160
yield R2 3.25 %
S3C_23013538
yield R2 3.31 %
S7D_469627544
yield R2 3.16 %
S2D_147451941
