# Supplementary material for: Genome‐wide association studies reveal genetic control of nutritional quality, milling traits, and agronomic characteristics in oat (Avena sativa L.)
Source: Plant Genome. 2025 Jul 7;18(3):e70060. doi: 10.1002/tpg2.70060 (PMC12234416; doi:10.1002/tpg2.70060)
Supplement: Supplementary file 7 [file TPG2-18-e70060-s008.pdf]

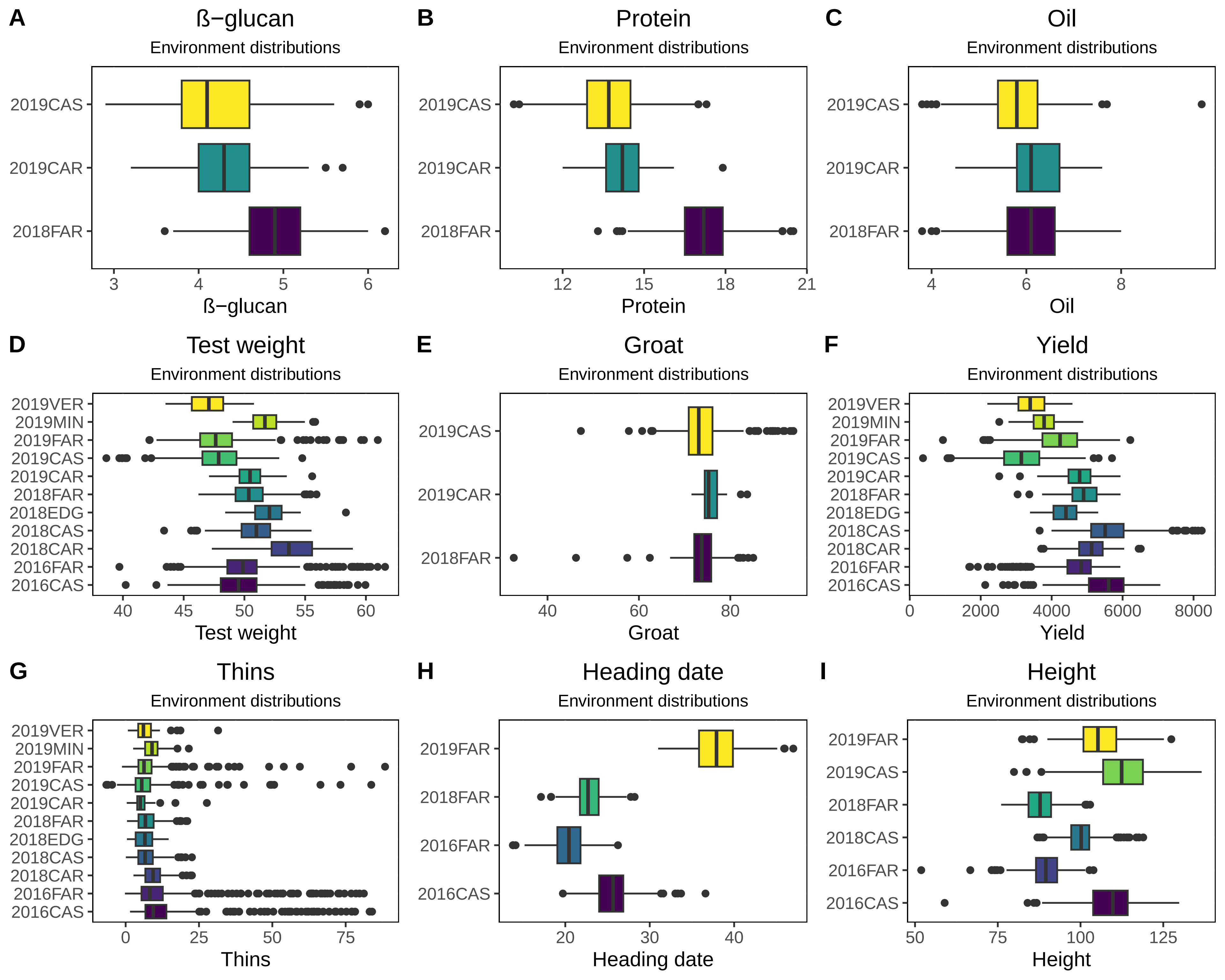
A
ß−glucan
Environment distributions
2019CAS
2019CAR
2018FAR
3
4
5
6
ß−glucan
B
Protein
Environment distributions
2019CAS
2019CAR
2018FAR
12
15
18
21
Protein
C
Oil
Environment distributions
2019CAS
2019CAR
2018FAR
4
6
8
Oil
D
Test weight
Environment distributions
2019VER
2019MIN
2019FAR
2019CAS
2019CAR
2018FAR
2018EDG
2018CAS
2018CAR
2016FAR
2016CAS
40
45
50
55
60
Test weight
E
Groat
Environment distributions
2019CAS
2019CAR
2018FAR
40
60
80
Groat
F
Yield
Environment distributions
2019VER
2019MIN
2019FAR
2019CAS
2019CAR
2018FAR
2018EDG
2018CAS
2018CAR
2016FAR
2016CAS
0
2000
4000
6000
8000
Yield
G
Thins
Environment distributions
2019VER
2019MIN
2019FAR
2019CAS
2019CAR
2018FAR
2018EDG
2018CAS
2018CAR
2016FAR
2016CAS
0
25
50
75
Thins
H
Heading date
Environment distributions
2019FAR
2018FAR
2016FAR
2016CAS
20
30
40
Heading date
I
Height
Environment distributions
2019FAR
2019CAS
2018FAR
2018CAS
2016FAR
2016CAS
50
75
100
125
Height
