# Supplementary figures and images for: Genome‐wide association studies reveal genetic control of nutritional quality, milling traits, and agronomic characteristics in oat (Avena sativa L.)
Source: Plant Genome. 2025 Jul 7;18(3):e70060. doi: 10.1002/tpg2.70060 (PMC12234416; doi:10.1002/tpg2.70060)

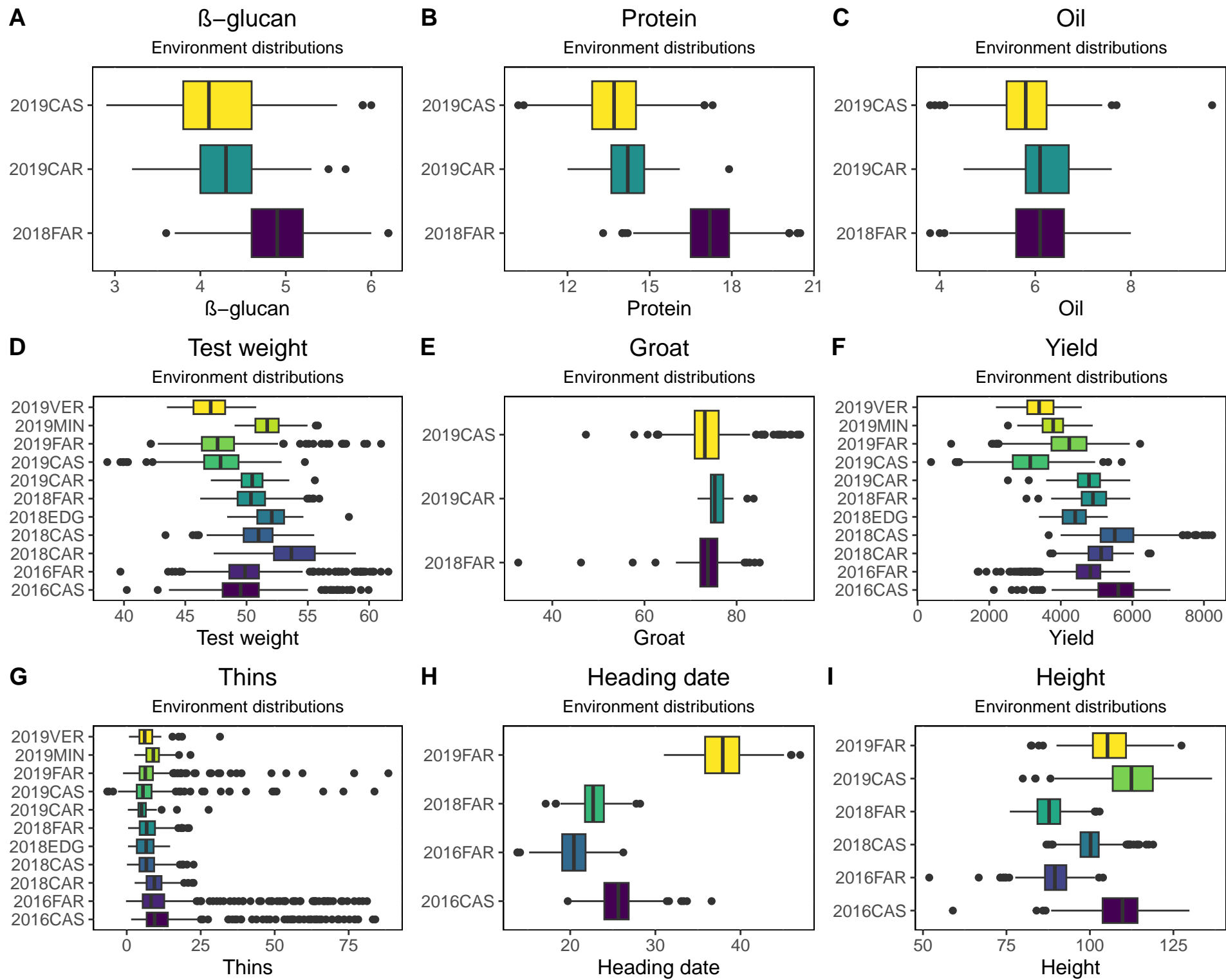

Supplement: Supplementary file 2 — Supplementary Figure 1 contains the distribution of spatially adjusted environment means for β‐Glucan (A), Protein (B), Oil (C), Test weight (D), Groat (E), Yield (F), Thins (G), Heading date (H) and Height (I). [file TPG2-18-e70060-s008.pdf]

PCA Plot

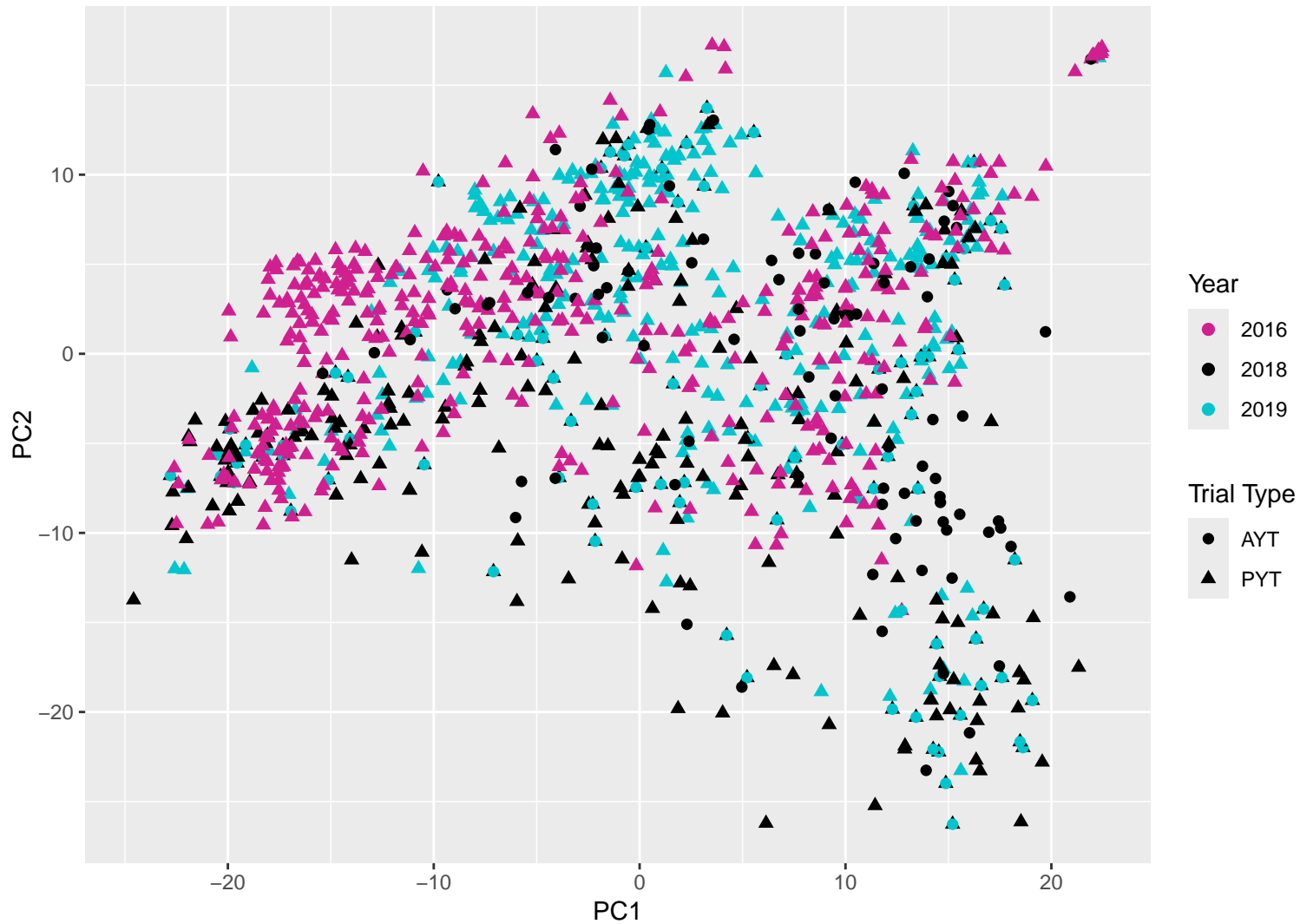

Supplement: Supplementary file 3 — Supplementary Figure 2 contains the principal component analysis of the taxa used in this study. [file TPG2-18-e70060-s004.pdf]

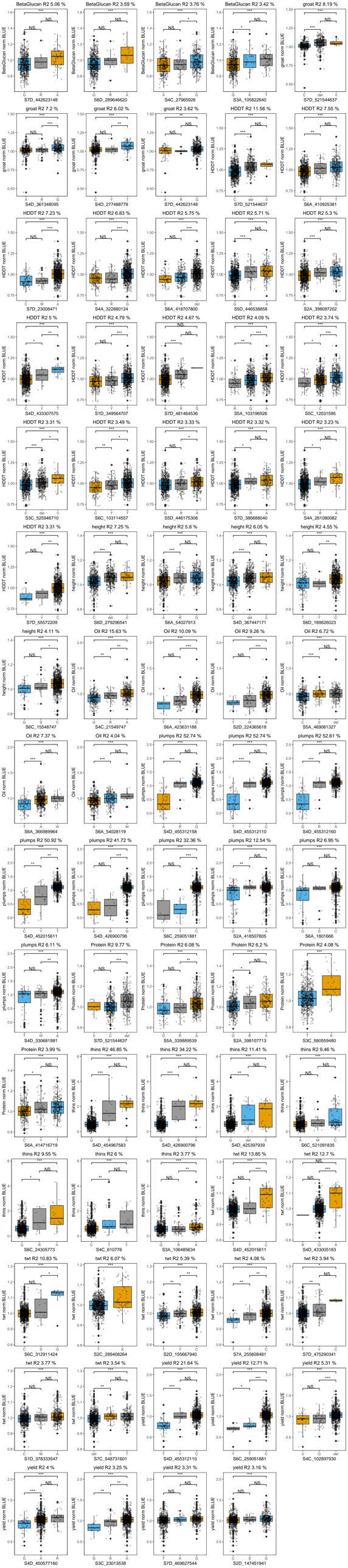

Supplement: Supplementary file 5 — Supplementary Figure 4 contains the distribution of normalized BLUE values grouped by allele state. [file TPG2-18-e70060-s007.pdf]

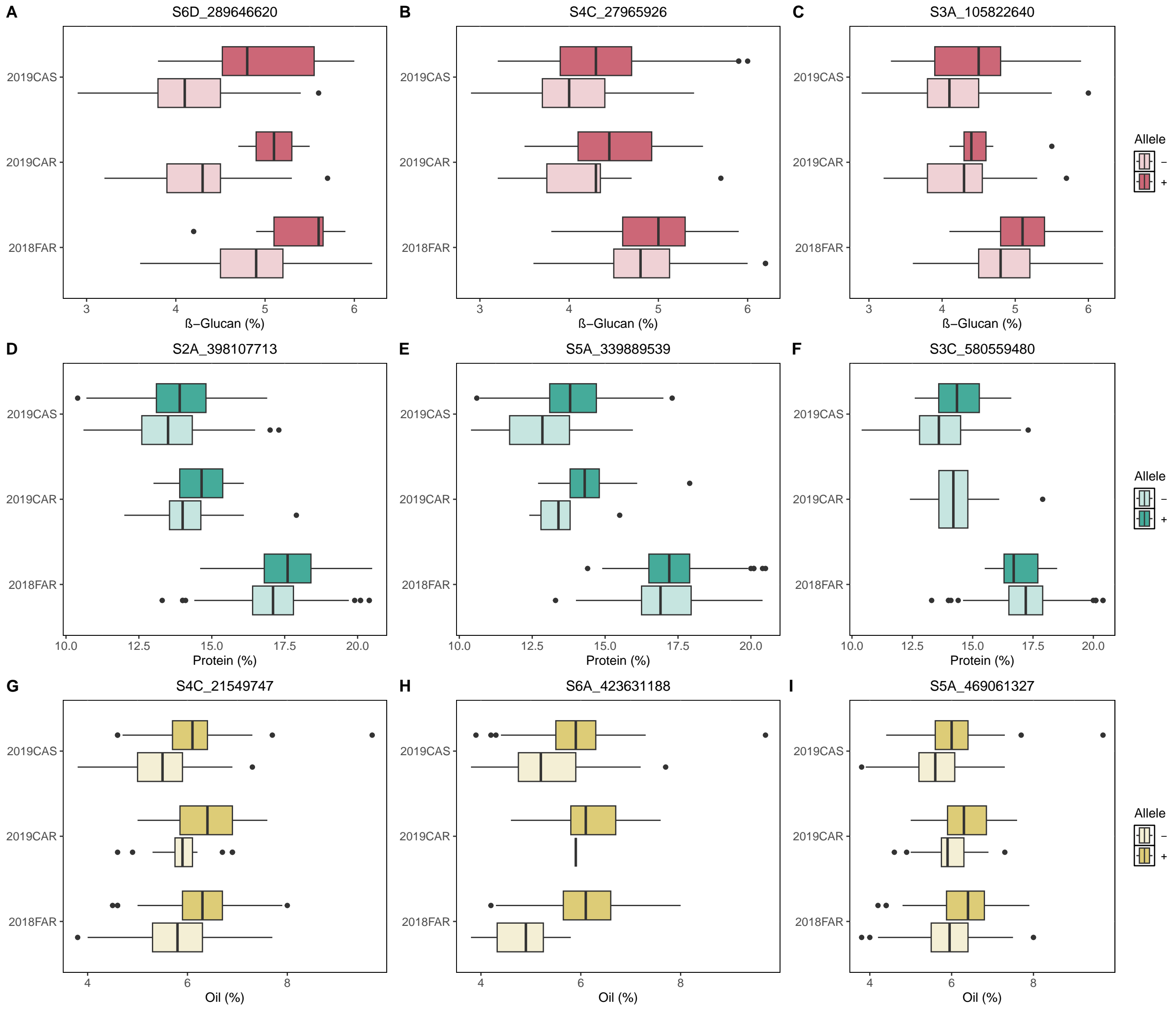

Supplement: Supplementary file 6 — Supplementary Figure 5 contains the distribution of spatially adjusted environment means grouped by the three most significant QTL for β‐Glucan (A‐C), Protein (D‐F), and Oil (G‐I). [file TPG2-18-e70060-s005.pdf]

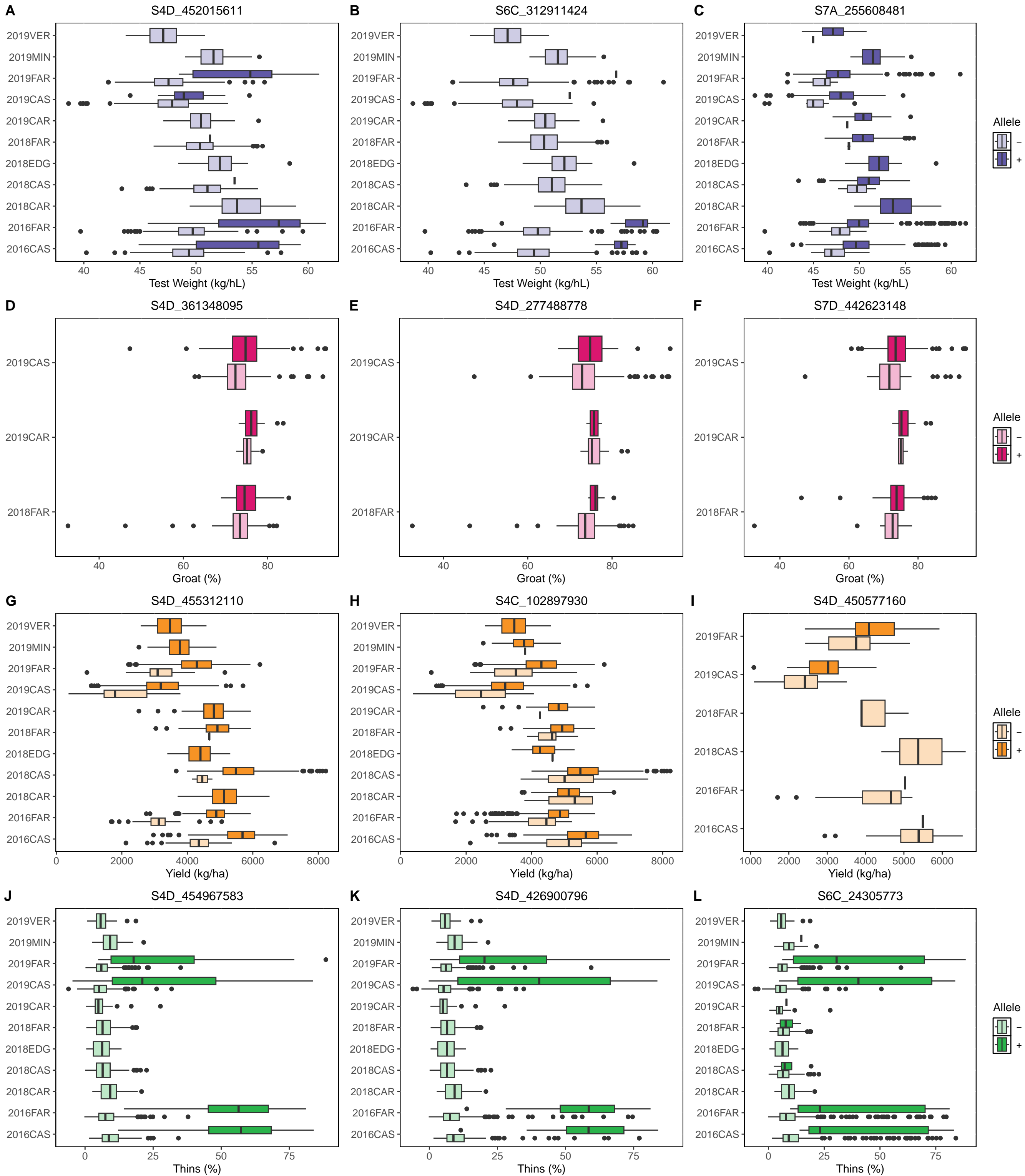

Supplement: Supplementary file 7 — Supplementary Figure 6 contains the distribution of spatially adjusted environment means grouped by the three most significant QTL for Test weight (A‐C), Groat (D‐F), Yield (G‐I), and Thins (J‐L). [file TPG2-18-e70060-s003.pdf]

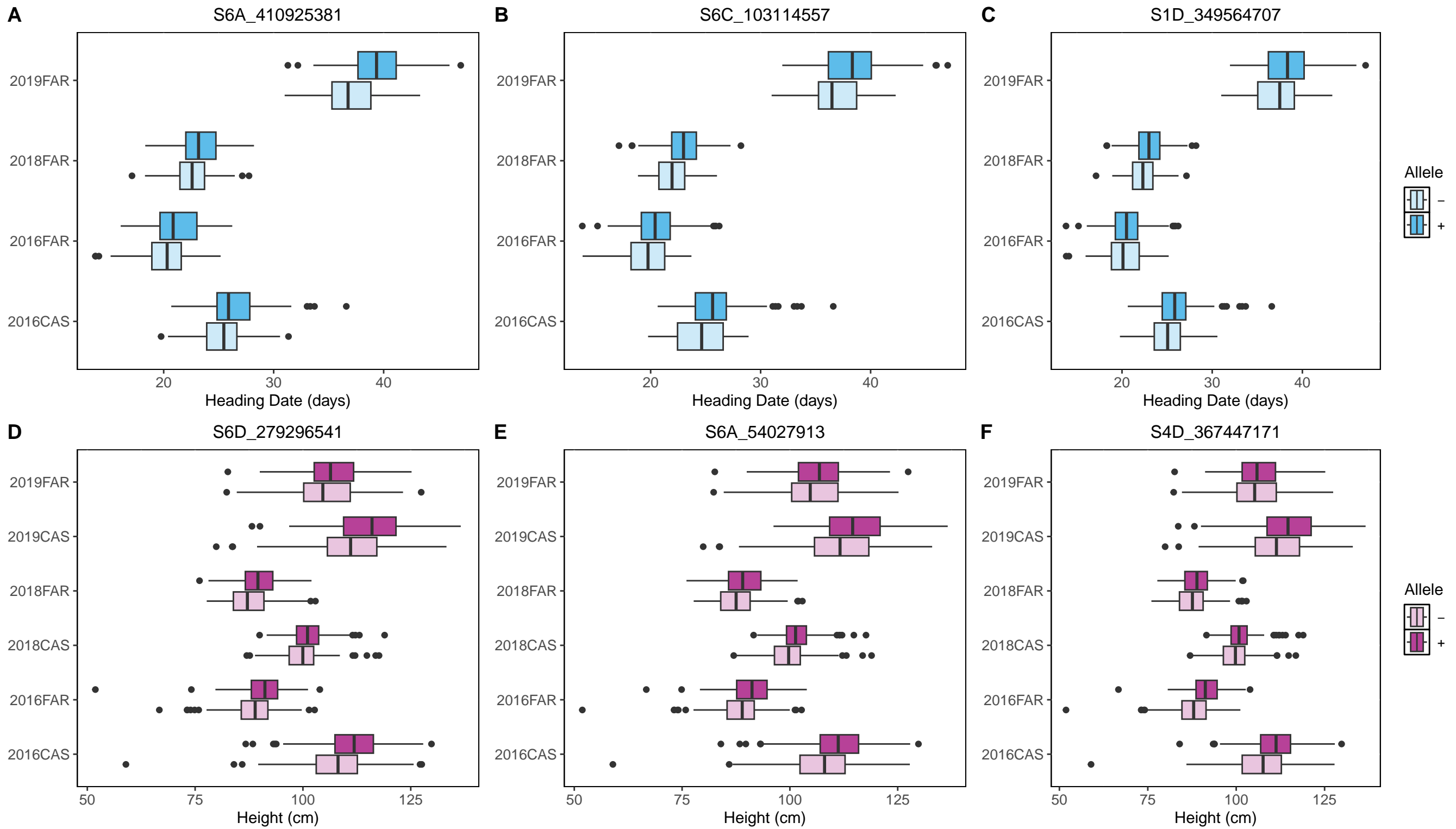

Supplement: Supplementary file 8 — Supplementary Figure 7 contains the distribution of spatially adjusted environment means grouped by the three most significant QTL for Heading Date (A‐C) and Height (D‐F). [file TPG2-18-e70060-s002.pdf]
