# Supplementary material for: Genome‐wide association studies reveal genetic control of nutritional quality, milling traits, and agronomic characteristics in oat (Avena sativa L.)
Source: Plant Genome. 2025 Jul 7;18(3):e70060. doi: 10.1002/tpg2.70060 (PMC12234416; doi:10.1002/tpg2.70060)
Supplement: Supplementary file 4 — Supplementary Figure 3 contains the GWAS results visualized with QQ and Manhattan plots for β‐Glucan (A,B), Protein (C,D), Oil (E,F), Test weight (G,H), Groat (I,J), Yield (K,L), Thins (M,N), Heading date (O,P) and Height (Q,R). [file TPG2-18-e70060-s006.pdf]

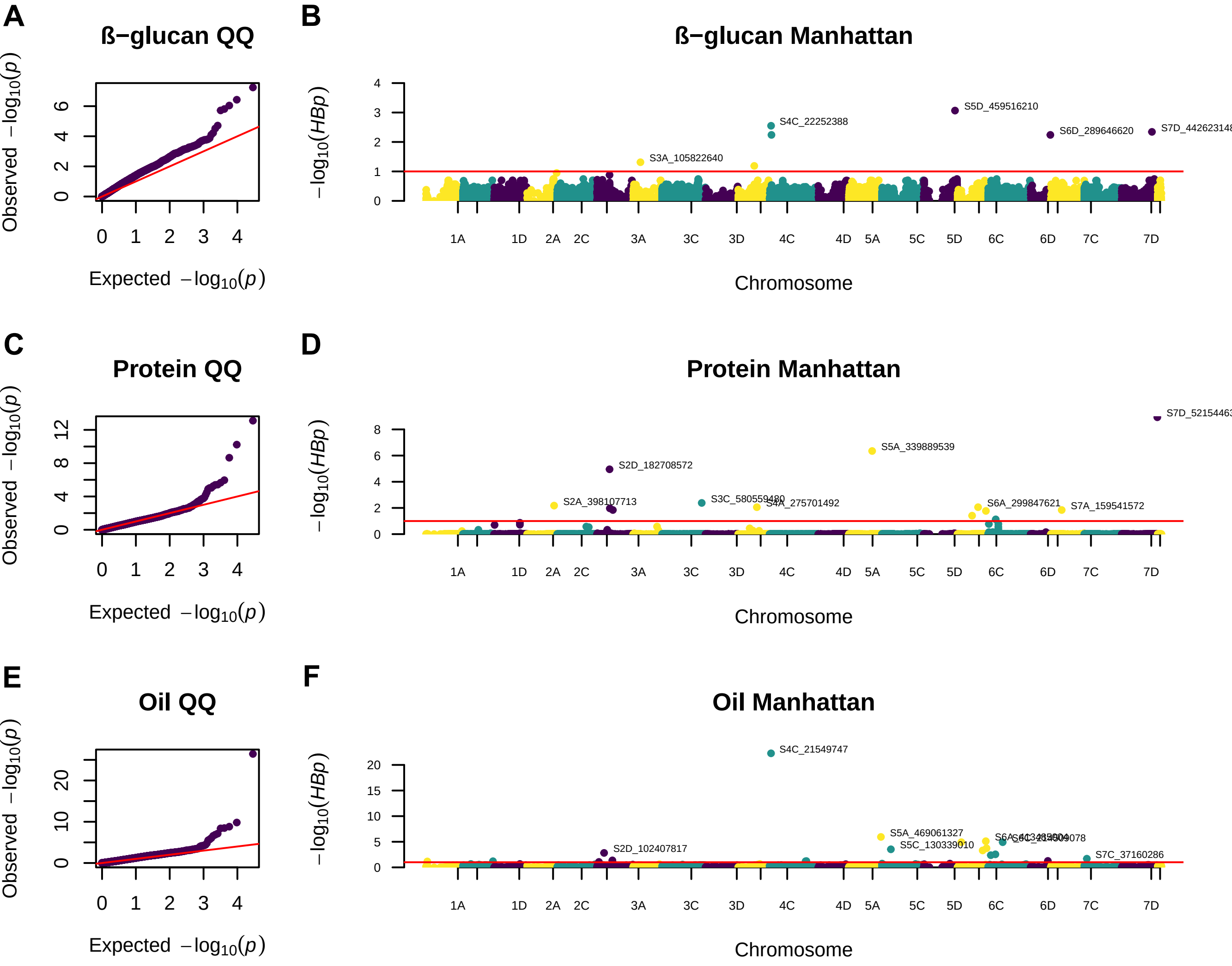

A
ß−glucan QQ
Observed −log10(p)
Expected −log10(p)
B
ß−glucan Manhattan
−log10(HBp)
S3A_105822640
S4C_22252388
S5D_459516210
S6D_289646620
Chromosome
1A 1D 2A 2C 3A 3C 3D 4C 4D 5A 5C 5D 6C 6D 7C 7D
C
Protein QQ
Observed −log10(p)
Expected −log10(p)
D
Protein Manhattan
−log10(HBp)
S2A_398107713
S2D_182708572
S3C_580559480
S4A_275701492
S5A_339889539
S6A_299847621
S7A_159541572
Chromosome
1A 1D 2A 2C 3A 3C 3D 4C 4D 5A 5C 5D 6C 6D 7C 7D
E
Oil QQ
Observed −log10(p)
Expected −log10(p)
F
Oil Manhattan
−log10(HBp)
S4C_21549747
S2D_102407817
S5A_469061327
S5C_130339010
S7C_37160286
Chromosome
1A 1D 2A 2C 3A 3C 3D 4C 4D 5A 5C 5D 6C 6D 7C 7D

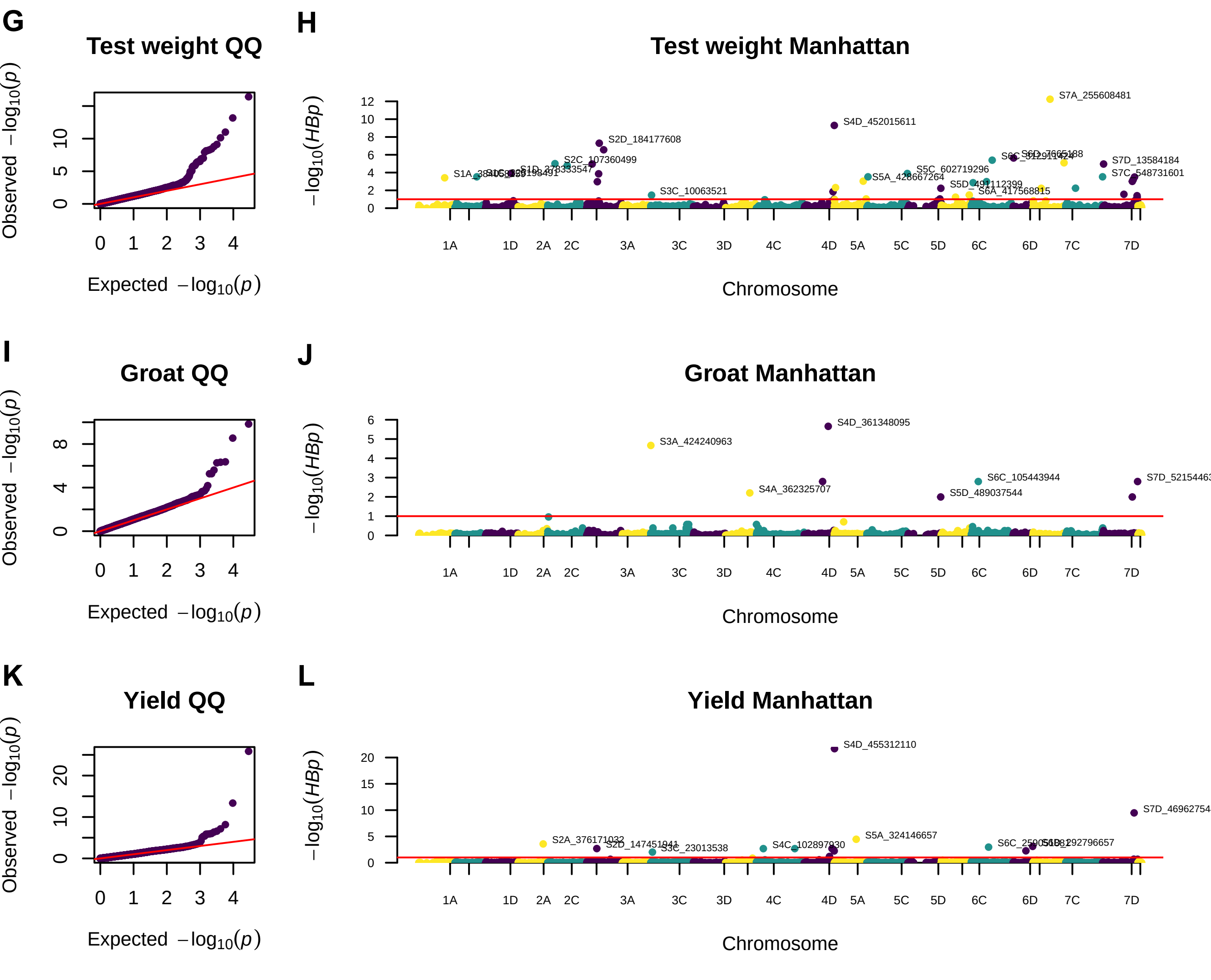

G
Test weight QQ
Observed −log10(p)
Expected −log10(p)
H
Test weight Manhattan
−log10(HBp)
Chromosome
S7A_255608481
S4D_452015611
S2D_184177608
S2C_107360499
S1A_38405
S1D_278333547
S3C_10063521
S5C_602719296
S5A_428667264
S5D_491112399
S6A_417568815
S7D_13584184
S7C_548731601
1A
1D
2A
2C
3A
3C
3D
4C
4D
5A
5C
5D
6C
6D
7C
7D
I
Groat QQ
Observed −log10(p)
Expected −log10(p)
J
Groat Manhattan
−log10(HBp)
Chromosome
S4D_361348095
S3A_424240963
S4A_362325707
S6C_105443944
S5D_489037544
K
Yield QQ
Observed −log10(p)
Expected −log10(p)
L
Yield Manhattan
−log10(HBp)
Chromosome
S4D_455312110
S7D_46962754
S5A_324146657
S2A_376171032
S3C_23013538
S4C_102897930

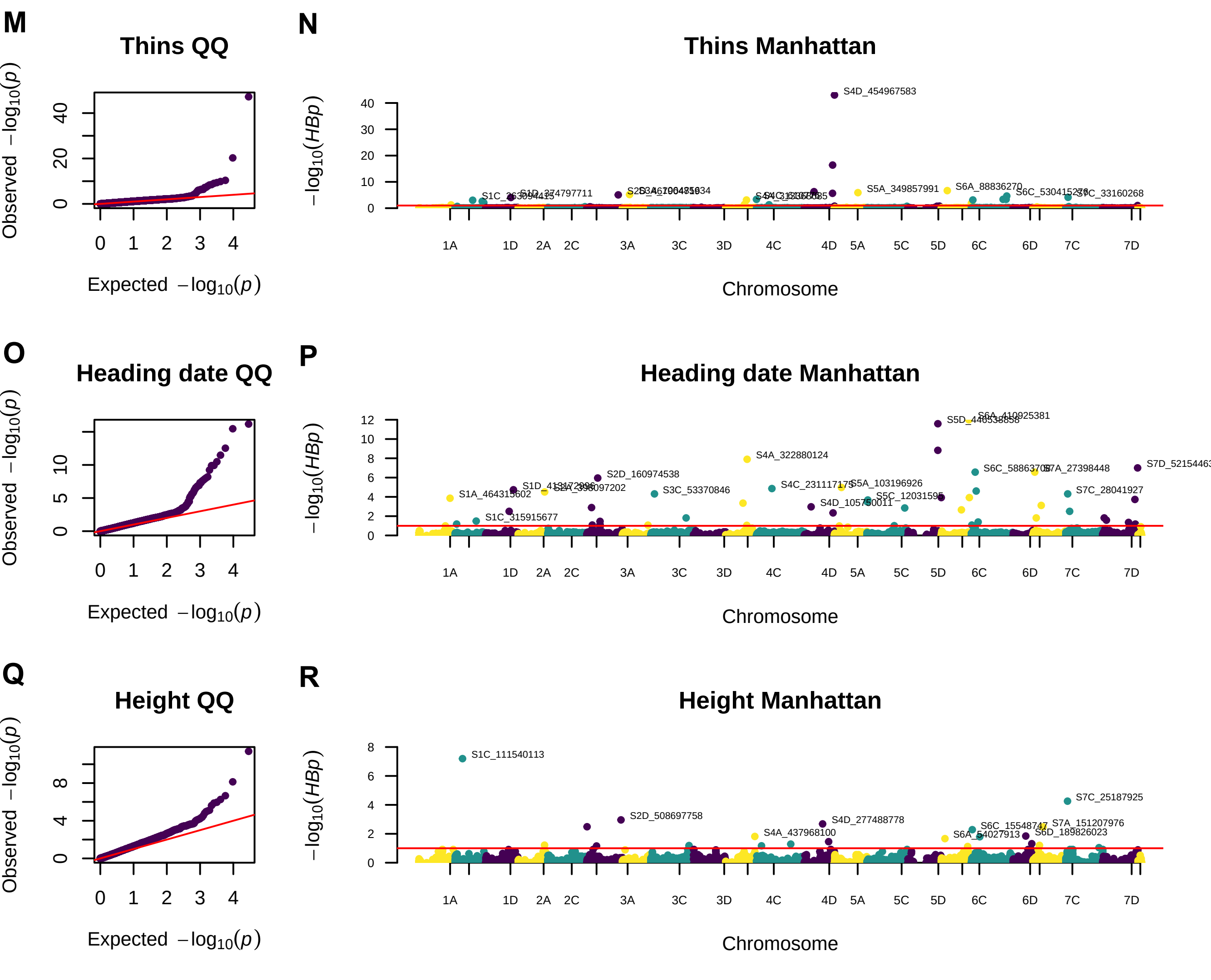

M
Thins QQ
Observed −log10(p)
Expected −log10(p)
N
Thins Manhattan
−log10(HBp)
S4D_454967583
S1C_263094415
S1D_374797711
S5A_349857991
S6A_88836270
S6C_530415278
S7C_33160268
Chromosome
1A
1D
2A
2C
3A
3C
3D
4C
4D
5A
5C
5D
6C
6D
7C
7D
O
Heading date QQ
Observed −log10(p)
Expected −log10(p)
P
Heading date Manhattan
−log10(HBp)
S6A_410925381
S4A_322880124
S2D_160974538
S6C_58863708
S7A_27398448
S4C_231117175
S5A_103196926
S1A_464315602
S3C_53370846
S5C_12031595
S7C_28041927
S4D_105750011
S1C_315915677
Chromosome
Q
Height QQ
Observed −log10(p)
Expected −log10(p)
R
Height Manhattan
−log10(HBp)
S1C_111540113
S7C_25187925
S2D_508697758
S4D_277488778
S4A_437968100
S6C_15548747
S7A_151207976
S6A_54027913
S6D_189826023
Chromosome
